# Slit-Dependent Endocytic Trafficking of the Robo Receptor Is Required for Son of Sevenless Recruitment and Midline Axon Repulsion

**DOI:** 10.1371/journal.pgen.1005402

**Published:** 2015-09-03

**Authors:** Rebecca K. Chance, Greg J. Bashaw

**Affiliations:** Department of Neuroscience, Perelman School of Medicine, University of Pennsylvania, Philadelphia, Pennsylvania, United States of America; Max-Planck Institute of Neurobiology, GERMANY

## Abstract

Understanding how axon guidance receptors are activated by their extracellular ligands to regulate growth cone motility is critical to learning how proper wiring is established during development. Roundabout (Robo) is one such guidance receptor that mediates repulsion from its ligand Slit in both invertebrates and vertebrates. Here we show that endocytic trafficking of the Robo receptor in response to Slit-binding is necessary for its repulsive signaling output. Dose-dependent genetic interactions and *in vitro* Robo activation assays support a role for Clathrin-dependent endocytosis, and entry into both the early and late endosomes as positive regulators of Slit-Robo signaling. We identify two conserved motifs in Robo’s cytoplasmic domain that are required for its Clathrin-dependent endocytosis and activation *in vitro*; gain of function and genetic rescue experiments provide strong evidence that these trafficking events are required for Robo repulsive guidance activity *in vivo*. Our data support a model in which Robo’s ligand-dependent internalization from the cell surface to the late endosome is essential for receptor activation and proper repulsive guidance at the midline by allowing recruitment of the downstream effector Son of Sevenless in a spatially constrained endocytic trafficking compartment.

## Author Summary

The formation of sterotyped neuronal connections during embryonic development is essential for animal survival and behavior. In particular, establishing proper connectivity at the midline is critical for the orchestration of rhythmic behaviors. Conserved genetic programs that instruct axon guidance at the midline have been characterized in multiple model systems, but the signaling mechanisms underlying axon guidance are not well understood. Slits and Robos comprise conserved families of axon guidance cues and receptors that control midline guidance by preventing inappropriate midline crossing. Here, we identify a novel mechanism that is required for Robo receptor activation and Robo-dependent axon repulsion *in vivo*. Using a combination of molecular genetic and cell biological approaches, we define a role for Slit-dependent trafficking of Robo from the plasma membrane to the early and late endosomes that contributes to Robo activation and signaling. In previous work, endocytic trafficking has been shown to modulate axon guidance responses by regulating surface levels of guidance receptors. In contrast, our observations indicate that endocytosis of the Robo receptor itself is required for receptor activation and precedes the recruitment of a key downstream signaling effector to the Robo receptor cytoplasmic domain.

## Introduction

The complex wiring patterns of the adult central nervous system are established by the stepwise navigation of growth cones and migrating cells through a series of choice points during development. At each choice point, the complement of guidance receptors expressed on the growth cone’s plasma membrane determines which of the cues in the extracellular environment will inform the cell’s guidance decision as it navigates toward its eventual synaptic partner. Understanding how an individual growth cone deploys its guidance receptors to make specific guidance decisions is critical to learning how proper wiring is established in development.

Roundabout (Robo) receptors comprise a family of highly conserved axon guidance receptors that mediate repulsion in response to their Slit ligands during neuronal development [1–4]. Robo receptors have also been implicated in genome-wide association studies with the pathogenesis of several human diseases including autism and schizophrenia [5,6], and they are thought to be causatively linked to dyslexia and periventricular nodular heterotopia [7], suggesting roles in guidance of more diverse axonal projections in the human cortex that are yet to be characterized.

In both invertebrates and vertebrates, Slits serve as repulsive cues to their Robo receptors by demarcating regions into which axons cannot maintain their exploratory projections. In the case of the *Drosophila* embryonic ventral nerve cord (VNC), Slit is expressed by midline glia, which creates a barrier for axonal projection for any growth cones expressing Robo at their surface [2,3]. In *robo* mutants normally ipsilaterally-projecting (ipsilateral or post-crossing commissural) axons ignore the presence of this repulsive cue and project into the midline and even circle there in namesake roundabouts [8]. Slit-mediated repulsive guidance can also instruct axonal projections by corralling fascicles into relative valleys of Slit expression- mouse callosal axons project between the indusium griseum and the glial wedge structures [9]- or by directing a 90° turn in bifurcating branches of sensory axons into the dorsal funiculus [10]. Analogously, there exists a relative valley in Slit expression in medio-lateral axis of the *Drosophila* VNC through which a sizeable set of longitudinal fascicles project [11].

The mechanism by which Slit triggers repulsion at the cellular level is not completely understood, but must involve an initial mis-projection into Slit-expressing regions in order to sense and then respond to the presence of the repulsive cue. One growth cone phenotype resulting from loss of Robo is defective filopodial retraction from the Slit-containing embryonic midline in *Drosophila*, resulting in stabilization of contralateral filopodial projections [12]. Similarly, loss of *robo2 (astray)* in zebrafish leads to abnormal stabilization of mis-projecting growth cones in the ventral forebrain, ‘errors’ that are normally corrected in wild-type [13]. The error-correction implicit in repulsive guidance from an initially adhesive protein-protein interaction requires some sort of physical severing which has been ascribed to juxtamembrane cleavage, endocytosis, or both [14–19].

Endocytosis in the growth cone has been implicated in the plasma membrane dynamics necessary for such responses as collapse[14,20,21], or, when applied asymmetrically, turning [22–24]. Endocytosis has also been implicated in the control over the complement of guidance receptors expressed on the growth cone surface, thereby fine-tuning sensitivity to extracellular cues [25–27]. Endocytic trafficking of Robo by Commissureless has also been demonstrated to negatively regulate delivery to the growth cone surface [28,29]. Endocytic trafficking of guidance receptors might serve not only to control surface receptor levels, but also to gate their activation once inside the cell. Evidence for this idea comes from the correlation between a requirement for the RhoGEFs *vav2* and *vav3* in both Ephrin endocytosis and proper retinogeniculate axon targeting [14], as well as the correlation between Rac activity in EphA receptor endocytosis and retinocollicular targeting [30]. Whether receptor endocytosis represents a general mechanism to control activation of repulsive guidance receptor signaling and whether the transit of internalized guidance receptors through distinct endocytic compartments is required for *in vivo* signaling is not known.

In this study, we identify Clathrin-dependent endocytosis of the Robo receptor as an obligate step in receptor activation and repulsive signaling. We present evidence that it is trafficking through endocytic compartments—following ligand-binding on the surface of the cell—that is required for receptor activation. We identify—with subcellular resolution–the early and late endosomes as compartments from which Robo signals, and identify the sequence motifs in Robo’s C-terminus that are required for its Slit-dependent internalization. Finally, we show that Slit-dependent endocytosis is required for both *in vitro* recruitment of the Ras/Rho GEF Son of Sevenless (Sos), a downstream effector of Robo repulsive signaling and for Robo-mediated midline repulsion *in vivo*.

## Results

### Endocytic trafficking genes genetically interact with *slit and robo*

**Fig 1:**
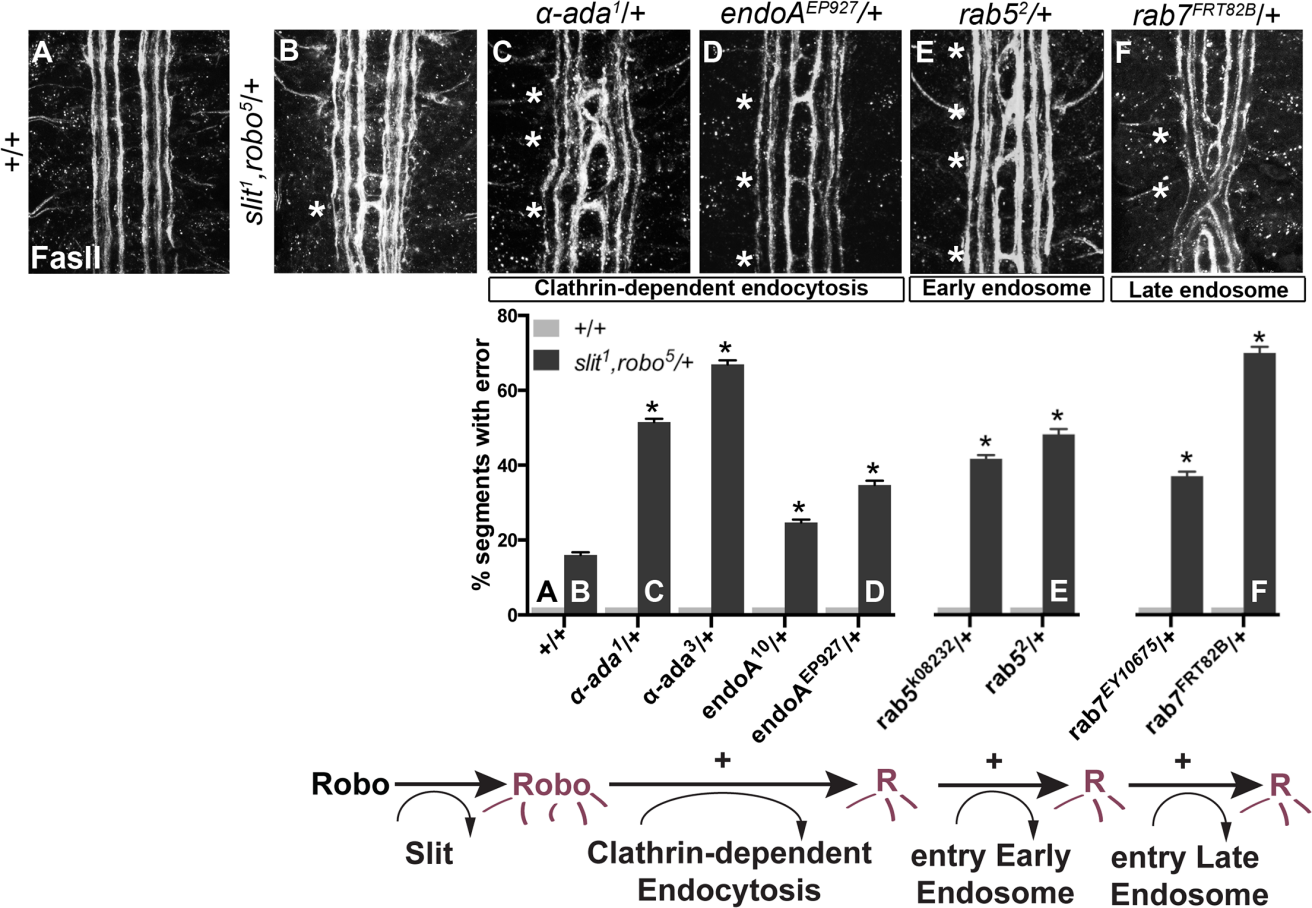
Genetic interactions between Clathrin-dependent endocytosis, and endocytic trafficking genes, and *slit* and *robo*. (A) An ipsilateral subset of axons in the ventral nerve cord of *WT* stage 16 *Drosophila* embryos are stained with a monoclonal antibody (mAb) to FasciclinII (FasII), and quantified in the histogram below as having 0% error in the number of embryonic segments with fascicles crossing the midline. (B) Double heterozygous *slit*, *robo* embryos have a mild loss-of-repulsion phenotype (induction of ectopic crossing events in 16% of embryonic segments). Inhibiting Clathrin-dependent endocytosis by removing one copy of either *α-adaptin* or *endophilinA* in the *slit*,*robo/+* background enhances the number of crossing defects (C, D), as does inhibiting either entry into the early endosome by removing one copy of *rab5* (E), or entry into the late endosome by removing one copy of *rab7* (F). These genetic enhancements of the *slit*,*robo/+*, but not of the *+/+*, ectopic crossing frequency are statistically significant (*, indicates p<0.0001) by two-way ANOVA, Sidak’s 95% Confidence Interval. Error bars indicate standard error of the mean. (*+/+* n (number of segments) = 121, *slit* ^*1*^,*robo* ^*5*^/+: 132; *α-ada* ^*1*^/+ 121, *α-ada* ^*1*^/ *slit* ^*1*^,*robo* ^*5*^ 99; *α-ada* ^*3*^/+ 121, *α-ada* ^*3*^/ *slit* ^*1*^,*robo* ^*5*^ 154; endoA^*10*^/+ 121, *slit* ^*1*^,*robo* ^*5*^/+; endoA^*10*^/+ 154; endoA^*EP927*^/+ 121, *slit* ^*1*^,*robo* ^*5*^/+; endoA^*EP927*^/+ 121; *rab5* ^*k08232*^/+ 121, *slit* ^*1*^,*robo* ^*5*^/*rab5* ^*k08232*^ 154; *rab5* ^*2*^/+ 121, *slit* ^*1*^,*robo* ^*5*^/*rab5* ^*2*^ 121; rab7^*EY10675*^/+ 121, *slit* ^*1*^,*robo* ^*5*^/+; rab7^*EY10675*^/+ 176; rab7^*FRT82B*^/+ 121, *slit* ^*1*^,*robo* ^*5*^/+; rab7^*FRT82B*^/+ 154.)

Based on previous findings suggesting a role for endocytosis in modulating axon guidance receptor activity and signaling, we could envision at least two plausible models for how Robo receptor endocytosis might regulate axon repulsion. If endocytosis modulates the amount of Robo receptor on the surface of the growth cone, a reduction in receptor endocytosis would be predicted to lead to increased levels of surface receptor and more robust repulsive signaling. Alternatively, if Robo receptor endocytosis is an obligate step in receptor activation, preventing or reducing Robo endocytosis would result in impaired repulsive signaling. To test which, if either, of these functions endocytosis might contribute to Slit-Robo signaling, we first sought genetic evidence implicating endocytic trafficking in midline axon repulsion. We examined an ipsilateral subset of axons whose projection patterns depend on Robo’s repulsive response to Slit. In *robo* mutants the medial-most of the FasII-positive fascicles invariably collapse and circle at the midline. Reducing *slit* and *robo* gene dose by half in heterozygous *slit*, *robo*/+ embryos results in a partial loss of repulsion, which represents a sensitized background in which we can detect both suppressors and enhancers (Fig 1). We, and others, have used this sensitized genetic background to uncover additional genes that contribute to midline repulsion [31–34]. In addition to offering a sensitive readout for alterations in midline repulsion, this strategy allows us to detect dominant genetic interactions, which avoids potential complications from removing all endocytic gene function, which would be predicted to have broad and early developmental defects.

**Fig 2:**
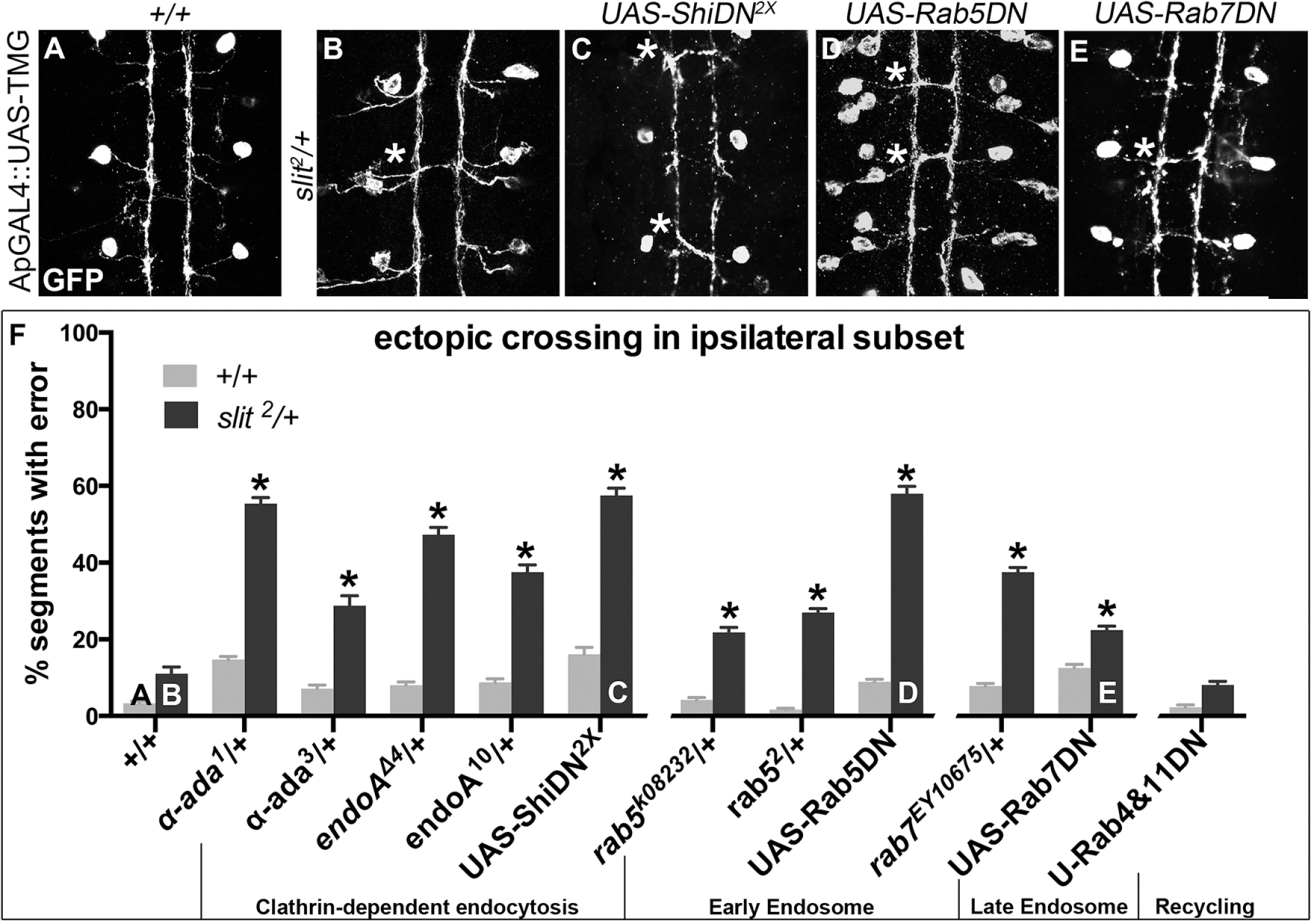
Genetic interactions between Dominant-Negative Transgenes for Clathrin-dependent endocytosis, and endocytosis through the late endosome, and *slit*. A more restricted ipsilateral subset of axons are genetically labeled with Tau-Myc-GFP transgene to highlight their microtubules and therefore axonal projection patterns. (A) In stage 16 WT embryos the two Ap axon fascicles on either side of the midline project ipsilaterally in all embryonic segments (3 shown here, 8 abdominal scored). (B) In animals where one copy of *slit* has been removed, a partial loss of repulsion phenotype results with 11% of segments exhibiting ectopic crossing events (indicated by *). (C) Inhibiting Clathrin-dependent endocytosis in a WT background by adding in Dominant-Negative (DN) transgenes to Shibire, the fly homolog to Dynamin, causes ectopic crossing errors in the Ap axons. (D) Inhibiting entry into the early endosome by expressing DN-Rab5 Transgene causes ectopic crossing, which enhances the background level present in *slit* heterozygotes. (E) Inhibiting entry into the late endosome with DN-Rab7 transgene expression also causes loss of repulsion, which enhances the background level of crossing in *slit* heterozygotes. (F) Histogram: Inhibiting entry into the recycling endosome does not enhance the background crossing in *slit* heterozygotes. These genetic enhancements are statistically significant (*, indicates p<0.0001)) by two-way ANOVA, Sidak’s 95% Confidence Interval. Error bars indicate standard error of the mean. (*+/+* n (number of segments) = 112, *slit* ^*2*^/+: 104; *α-ada* ^*1*^/+ 320, *α-ada* ^*1*^/ *slit* ^*2*^ 112; *α-ada* ^*3*^/+ 112, *α-ada* ^*3*^/*slit* ^*2*^ 80; *endoA* ^*Δ4*^/+ 88, *slit* ^*2*^/+; *endoA* ^*Δ4*^/+ 112; endoA^*10*^/+136, *slit* ^*2*^/+; endoA^*10*^/+ 120; *UAS-ShiDN*/+;UAS-ShiDN/+ 112, *slit* ^*2*^/UAS-ShiDN; *UAS-ShiDN*/+ 80; *rab5* ^*k08232*^/+ 144, *slit* ^*2*^/*rab5* ^*k08232*^ 110; *rab5* ^*2*^/+ 232, *slit* ^*2*^/*rab5* ^*2*^ 152; UAS-Rab5DN/+ 168, *slit* ^*2*^/+; UAS-Rab5DN/+ 88; rab7^*EY10675*^/+ 128, *slit* ^*2*^/+; rab7^*EY10675*^/+ 112; UAS-Rab7DN/+ 128, *slit* ^*2*^/+; UAS-Rab7DN/+ 152; *UAS-Rab4DN*/+;UAS-Rab11DN/+ 128, *slit* ^*2*^/UAS-Rab4DN; *UAS-Rab11DN*/+ 136.) See also S1 Fig.

We screened mutants in known regulators of endocytosis for genetic interactions with *slit* and *robo*, including mutations in genes involved in (1) Clathrin-dependent endocytosis- *alpha-adaptin* and *endophilinA*-, (2) entry into the early endosome–*rab5*- and (3) entry into the late endosome- *rab7*. Removing one copy of *α-adaptin* and *endophilinA*- genes involved in cargo loading and formation of clathrin coated pits [35,36]–enhances the number of crossing errors compared to *slit*, *robo/+* heterozygotes (Fig 1B–1D). Removing one copy of either *rab5*, which regulates entry into the early endosome, or *rab7*, which regulates entry into the late endosome also enhances ectopic crossing (Fig 1E and 1F). In order to corroborate these findings, we tested for genetic interactions between the mutant alleles of endocytic trafficking genes and *slit* in another, more restricted subset of axons (Fig 2A). Just like the FasII+ subset of axons, the normally ipsilateral Apterous+ (Ap) axons are sensitive to partial loss of repulsion; a loss of one copy of *slit* alone induces ectopic crossing events in 11% of embryonic segments (Fig 2B). Inhibiting Clathrin-dependent endocytosis in this sensitized background by removing one allele of *α-adaptin* or *endophilinA* enhances the frequency of ectopic crossing events (Fig 2F). Removing one copy of *rab5* or *rab7* also enhances ectopic crossing errors. These genetic interactions suggest that trafficking from the plasma membrane, and into the early and late endosome positively regulate repulsive midline guidance. Together these observations are consistent with endocytosis contributing to receptor activation, as opposed to a modulation of surface levels available to bind Slit.

To determine whether the endocytic trafficking events relevant for midline guidance are occurring in neurons, we mis-expressed Dominant-Negative (DN) transgenes to inhibit components of the endocytosis machinery in the Ap neurons. Ectopic expression of DN forms of *shibire*, *Drosophila* Dynamin, (to block scission of invaginated Clathrin-coated pits [37,38]), Rab5 and Rab7 (to prevent entry into early and late endosomes, respectively), but not Rab4 and Rab11 (to prevent entry into the recycling endosome), results in enhancement of the ectopic crossing defects that are observed in *slit* heterozygotes (Fig 2C–2F). These findings are consistent with a model in which endocytic trafficking in neurons is contributing to Slit-Robo mediated repulsion. Further, the ectopic crossing events caused by expressing ShiDN or Rab5DN in the Ap axons in *slit* heterozygotes are fully rescued by increasing signaling of the Robo pathway by co-expression of a wild type Robo transgene (S1A Fig): an observation that is consistent with a specific requirement for endocytic regulation during Slit/Robo repulsion. Taken together, these data are consistent with a model in which endocytic trafficking from the plasma membrane into the early and late, but not the recycling endosome of neurons positively regulates Robo-mediated midline repulsion.

However these interactions alone cannot distinguish between the possibilities of endocytosis positively regulating repulsion from the midline, or negatively regulating attraction to the midline. We directly tested the latter hypothesis by assaying whether reducing the dosage of endocytic trafficking genes could enhance the ectopic crossing errors induced by enhanced midline attraction resulting from ectopic expression of the attractive guidance receptor Frazzled [39]. We detect no statistically significant difference between the observed crossing frequency and the predicted percentage crossing frequency from an additive interaction (S1B Fig), suggesting that endocytosis is not negatively regulating attractive guidance. These observations further support the interpretation that disrupting endocytosis is specifically affecting midline repulsion.

### Clathrin-dependent endocytosis from the cell surface through the early and late endosome positively regulate Robo signaling *in vitro*

Our genetic interaction data are consistent with endocytosis in neurons positively regulating Slit/Robo-mediated repulsive guidance, but they do not provide insight into the cell and molecular mechanism. In order to test whether this positive regulation of repulsive signaling is due to endocytosis of the Robo receptor itself, we assayed whether manipulations to Robo’s capacity to undergo endocytosis would affect its signaling. Using sequence alignment with known binding motifs to AP-2, the Clathrin adaptor complex expressed specifically on the surface of cells, we identified two tyrosine-based motifs in Robo’s C-terminus that are both conserved in human Robo1 sequence and predicted to be required for loading of Robo into Clathrin-coated pits—(1) YLQY, of the type YXXФ [40], and (2) YQAGL, like the tyrosine containing sorting signals in the epidermal growth factor receptor (EGFR) and L1/NgCAM [41,42] (S2J Fig). If Robo’s trafficking through the endocytic pathway is required for its repulsive response to Slit binding, then we would predict that both reducing Shibire function, and disrupting Robo’s ability to be loaded into Clathrin-coated pits would disrupt Robo signaling.

**Fig 3:**
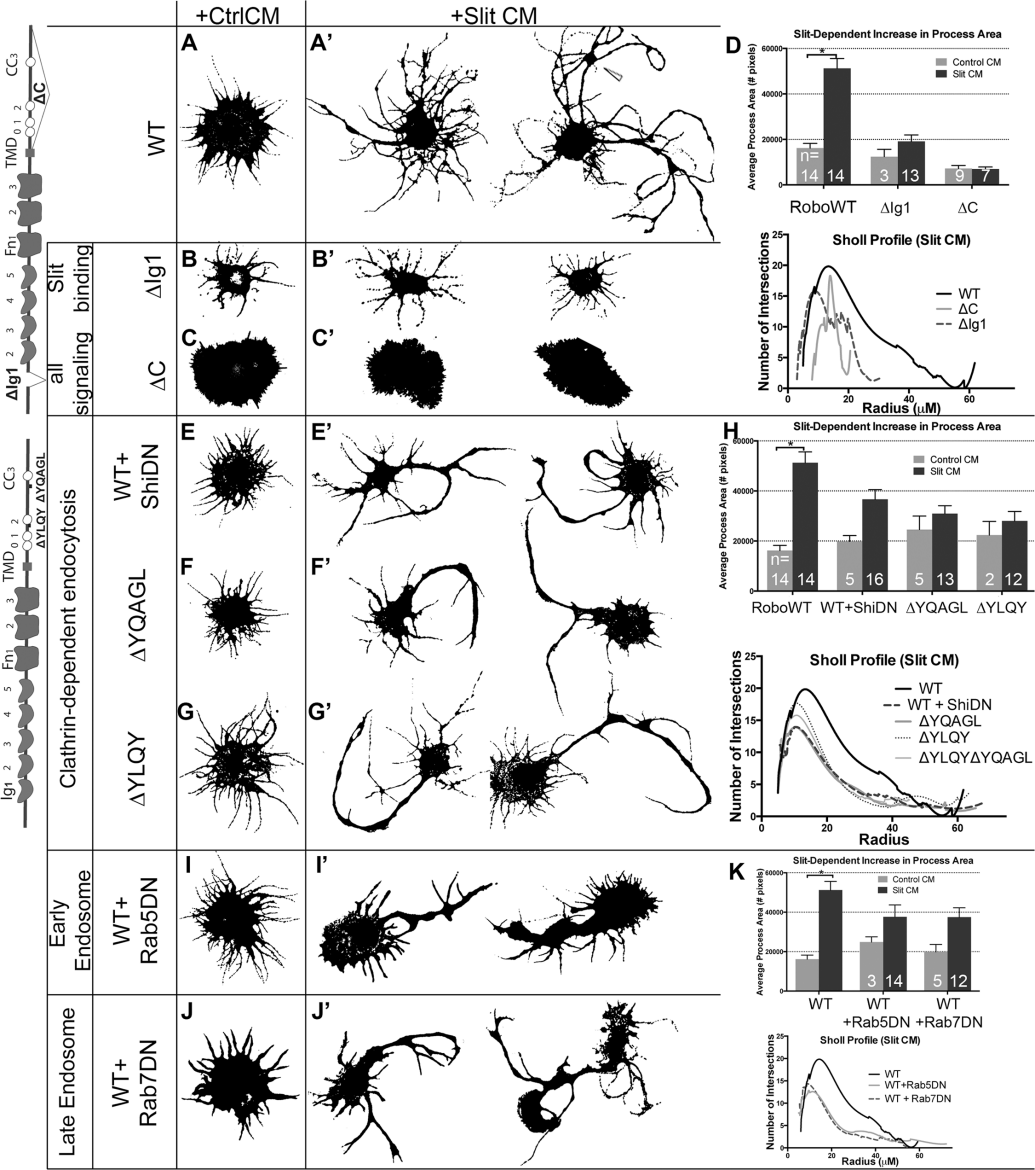
Clathrin-dependent endocytosis from the cell surface through the early and late endosome positively regulate Robo signaling *in vitro*. Morphological profiles of Drosophila embryonic cells bath treated for 10’ with Conditioned Media (CM) from cells either expressing empty vector (“Control”, A-C, E-G, I-J), or secreting Slit (A-C’, E-G’, I-J’). Cells expressing WT Robo that are treated with Control CM show a baseline level of process generation (A) that are more branched and elaborated if Slit treated, with two representative examples shown in (A’). This change is quantified as an increase in the average process area of multiple cells in the histogram, which is statistically significant by Two-way ANOVA, Sidak’s 95% Confidence Interval (n’s denoted on histogram). Error bars indicate standard error of the mean (D: n’s displayed on each bar). The Sholl analysis profile below reflects process field complexity as a function of the cells’ radii for cells treated with Slit CM (WT n = 13, ∆C n = 5, ∆Ig1 n = 8). (B) Cells that express Robo missing their Slit-binding motif (∆Ig1), do not elaborate processes in response to Slit treatment (B’), and show a smaller total process area, and a drop in the process field maximum radius in the Sholl profile (D). (C) Cells expressing Robo that lacks the ability to signal (∆C-terminus) show short processes that don’t branch or elaborate in response to Slit treatment (C’, D, E, E’). Inhibiting Clathrin-dependent endocytosis directly by cotransfection of WT Robo with DN-Shibire, the fly homolog of Dynamin, causes no change in the average process area of cells treated with Control CM but a defect in process elaboration in response to Slit treatment as compared to WT alone (A’), quantified as a decrease in the total process area in cells treated with Slit CM and a downward shift in the Sholl profile (H: ShiDN n = 13, ∆YQAGL n = 13, ∆YQAGL n = 12, ∆YLQY∆YQAGL n = 10). (F, G) Cells expressing Robo carrying deletions of either of two motifs predicted to be required for binding to AP-2, the Clathrin-adaptor complex expressed on the surface of cells, look similar to cells in (E’). (I, J) Inhibiting entry into the early (I’), or late (J’) endosome by co-expression of DN-Rab5, or DN-Rab7, respectively, with WT Robo causes a decrease in total process area, and a downward shift in the Sholl profile (K: Rab5DN n = 14, Rab7DN n = 13). See also S2 Fig.

To explore these possibilities, we developed an *in vitro* system to determine whether endocytosis of the Robo receptor can occur in response to Slit, and whether this process contributes to receptor signaling. *Drosophila* embryonic cells transfected with Robo that are bath treated for 10 minutes with Slit-conditioned media (CM) exhibit a robust spreading behavior, forming elaborate branched structures (Fig 3A). In contrast, cells transfected with Robo and treated with CM from cells expressing empty vector show no such response (Figs 3, S2B, S4A and S4B). We have quantified this spreading behavior in two ways- first, we compute the total area of each cells’ processes as a number of pixels, and, using representative cells, compute the Average Process Area as a function of transfected Robo and type of CM treatment (histogram, Fig 3D). To characterize the branching of processes in Slit-treated cells, we also performed Sholl analyses to compute the complexity of individual cell’s process field as a function of its radius starting after the cell cortex. These analyses are graphically displayed as the average Sholl profile of many cells treated with Slit CM (Fig 3D).

To assay whether the observed process elaboration behavior is indeed a readout of Robo activation in response to Slit we tested the following negative control variants of Robo: 1) deletion of the ectodomain (RoboΔEcto, S2A’ Fig), 2) deletion of the first immunoglobulin domain (RoboΔIg1, Fig 3B), the minimal region that interacts physically with Slit’s D2 domain [43–45], or 3) Robo missing its entire C-terminus (ΔC, Fig 3C), which is required for all signaling output [46]. Each of these mutated forms of Robo show a loss of process elaboration in response to Slit. We also noted that expression of Robo∆C results in variable increase in the size of the cell cortex even in the absence of Slit treatment; however, since the Slit-dependent branch elaboration that we observed and quantified is independent of effects on the cell cortex, we did not explore this phenomenon further. Robo that is missing its Conserved Cytoplasmic CC2 and CC3 motifs, required for binding of the downstream effectors Ena, Dock, Pak, SOS and therefore Rac activation [32,47,48], also display impaired spreading behavior (S2C Fig). These observations support the idea that Robo signaling in response to Slit binding is required for the Rac-dependent spreading behavior seen in WT Robo-expressing cells.

Next we wanted to test for a role for Clathrin-dependent endocytosis in Robo’s ability to generate branched processes in response to Slit treatment. We find that inhibiting endocytosis directly by co-transfection with DN Shibire (Fig 3E), or treatment with the Dynamin inhibitor Dynasore (S2D Fig), reduces the complexity of processes generated in response to Slit, as does deleting entirely, or point mutating the tyrosine residues of either of the two putative AP-2 binding motifs in Robo’s C-terminus (Figs 3F, 3G and S2F–S2H). Deleting both motifs at the same time also results in a smaller maximum radius of the process field (Figs 3H and S2E), similar to deleting the entire C-terminus, suggesting that the two AP-2 interacting motifs are each required for, and additively contribute to, Robo signaling. The qualitative and quantitative similarity in the process morphology of Slit-treated cells where Robo endocytosis is prevented, either by global disruption (Dynasore or DN Shibire) or by specific Robo mutations, suggests a contribution of receptor internalization to Robo’s activation. In addition, we find that endocytic trafficking, beyond internalization from the surface, through the early and late endosome also positively regulate Slit-dependent process elaboration. Inhibiting entry to the early or late endosome by co-expression of DN-Rab5 (Fig 3I), or DN-Rab7 (Fig 3J), respectively, also reduces branching complexity in Robo expressing Slit CM-treated cells. These data are consistent with a requirement for Clathrin-dependent endocytosis of the Robo receptor and trafficking into the early and late endosome for Slit-dependent process branching and outgrowth.

### Slit-dependent Robo removal from surface depends on C-terminal motifs

**Fig 4:**
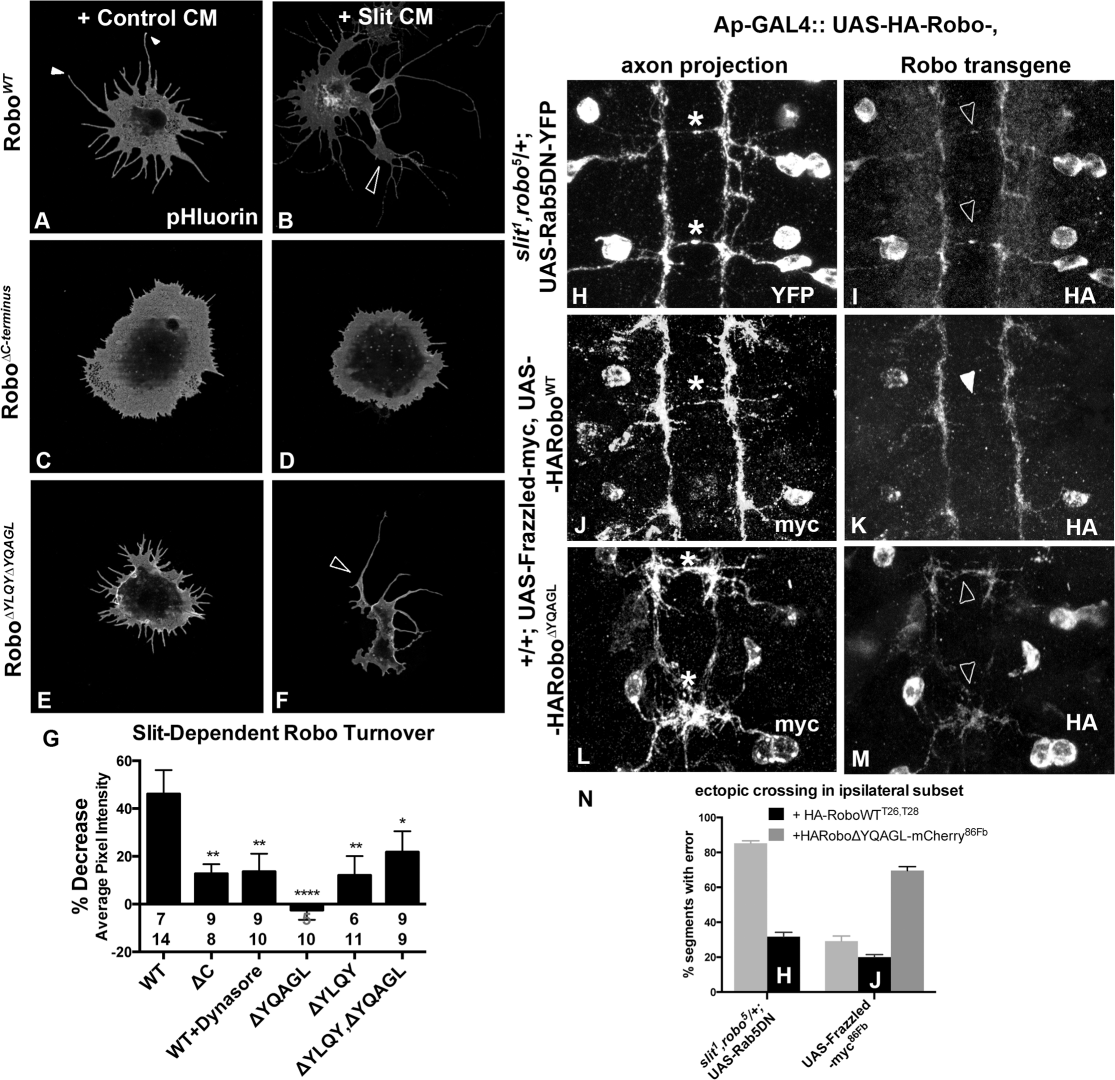
Clathrin-dependent Endocytosis is required for removal of Robo from the surface. An N-terminal pH sensitive tag on Robo (A-F) reveals the pool of Robo expressed on the surface of S2R+ cells after 2’ of conditioned media (CM) bath-treatment. S2R+ cells treated with CM from cells expressing Slit (B) as opposed to empty vector (A) show a decrease in surface levels of Robo, quantified in (G) as a percent decrease in average pixel intensity value of processes in (B) as compared to (A). (C, D) Inhibiting Robo signaling by deleting the entire C-terminus shunts the Slit-dependent reduction in average pixel intensity value of surface Robo, leading to a smaller percentage decrease in (G). (E, F) Deleting both of Robo’s putative AP-2 motifs abrogates the Slit-dependent reduction in surface receptor levels, leading to a smaller % decrease in average pixel intensity in (G), significant (*) according to one-way ANOVA Dunnett’s test (n’s for Control and Slit CM displayed below each bar). (H-M) The ectopic crossing events of a normally ipsilateral subset of axons in the ventral nerve cord of Stage 16 *Drosophila* embryos are induced by either manipulating entry to the early endosome with expression of DN-Rab5 transgene (H), or by overexpression of an attractive guidance receptor, Frazzled (J, L). Robo transgene is mislocalized to the ectopically crossing segments of axons in embryos defective for endocytic trafficking (I) but not in those with excessive attractive guidance (K), despite the similarity in strength of ectopic crossing phenotype (N). In contrast, Robo transgene defective for AP-2 binding is mislocalized to the ectopically crossing segments of axons (M) in the same gain of attraction background (L). See also S3 Fig.

In order to assess whether Robo’s C-terminal putative AP-2 interaction motifs indeed disrupt ligand-dependent endocytosis we directly assayed for a change in surface Robo levels in response to Slit in the same *in vitro* system. Using pHluorin, a pH sensitive GFP tag, on Robo’s N-terminus to distinguish surface Robo from the Robo protein in the lower pH environment of most cytosolic compartments, we analyzed the Slit-dependent reduction in surface receptor levels in S2R+ cells (Fig 4A–4F). In cells transfected with wild-type pHluorin–tagged Robo, there is a reduction in the fluorescence intensity of pHluorin in Slit-treated, as compared to control treated cells, which we quantified as a percent decrease in average signal intensity across many cells (Fig 4A, 4B and 4G). This Slit-dependent decrease in surface signal is inhibited by deleting Robo’s C-terminus (Fig 4C and 4D), suggesting a requirement for signaling in the Slit-dependent reduction in Robo surface levels. Evidence that our small deletions disrupt Clathrin-dependent endocytosis comes from the similarity of their effect on surface levels to the effect observed by inhibiting Shibire with the Dynamin inhibitor drug Dynasore [49]. In both cases the Slit-dependent decrease in surface Robo is prevented (Figs 4E, 4F and S3), consistent with Slit stimulating Clathrin-dependent endocytosis of Robo.

Analyzing trends in the spatial distribution of surface Robo intensity with reference to anatomical structures reveals clues about the mechanism of Robo internalization and branch formation. Tips of S2R+ processes bear peaks in surface Robo signal (closed arrowheads in Figs 4A and S3A), which is similar to Robo localization on the tips of filopodia in the developing fly embryo [3] and in primary *Drosophila* neuron cultures [50]. In the cells that have responded to Slit treatment by reducing their Robo surface levels, presumably by Clathrin-dependent internalization from the surface, process branch-points are marked by reduction in surface Robo levels (open arrowhead in Fig 4B). When inhibiting endocytosis, Robo signal stays high on both the processes with enlarged diameters and in the branch points that do exist (open arrowhead Fig 4F), likely due to lack of Slit-dependent internalization. The correlation between the absence of receptor internalization, either by globally inhibiting endocytosis with Dynasore (S4A and S4B Fig), or by deleting or point-mutating AP-2 adaptor motifs in Robo’s C-terminus (Figs 4E–4G and S3C–S3G), and decreased process elaboration (Figs 3H and S3F–S3H) suggests that Clathrin-dependent endocytosis of Robo is required for its signaling output.

To test whether the link between endocytic trafficking and Robo signaling is also observed *in vivo*, we analyzed Robo distribution and midline guidance in the embryo. The endogenous expression pattern of Robo throughout the embryonic ventral nerve cord is characterized by commissural exclusion and longitudinal enrichment [3]. If endocytic trafficking of Robo is required for repulsive signaling, we would expect to see a correlation between Robo mislocalization and guidance errors in embryos with defective endocytic trafficking. In fact, when we induce guidance errors by manipulating entry into the early endosome by expressing DN-Rab5 (asterisks, Fig 4H), we see mislocalization of Robo to the ectopically midline projecting segments of normally ipsilateral axons (open arrowheads, Fig 4I). This correlation between Robo mislocalization and guidance errors is specific to endocytic trafficking manipulations; when we induce ectopic crossing events by overexpressing the Frazzled attractive guidance receptor (asterisk, Fig 4J), we find no mislocalized Robo on the crossing portions of axons, despite the similar number of ectopic crossing events (closed arrowhead, Fig 4L). Further, Robo missing its AP-2 adaptor motif is also mislocalized to the commissural segments (open arrowheads Fig 4M) of ectopically crossing axons (asterisks, Fig 4L). Finally, Robo is mislocalized to the collapsed Ap axon fascicles in embryos deficient for Slit, and to the ectopically crossing portions of axons in *slit*, *robo/+* double heterozygotes expressing Robo missing its Slit-binding domain (S3H Fig). Taken together these data suggest that Slit stimulates endocytosis of the Robo receptor, and that this decrease in surface signal is required for receptor signaling in the receiving cell as evidenced by the reduction in process elaboration in S2R+ cells and midline guidance errors *in vivo*.

### Slit induces Robo colocalization with the early endosomal marker Rab5

**Fig 5:**
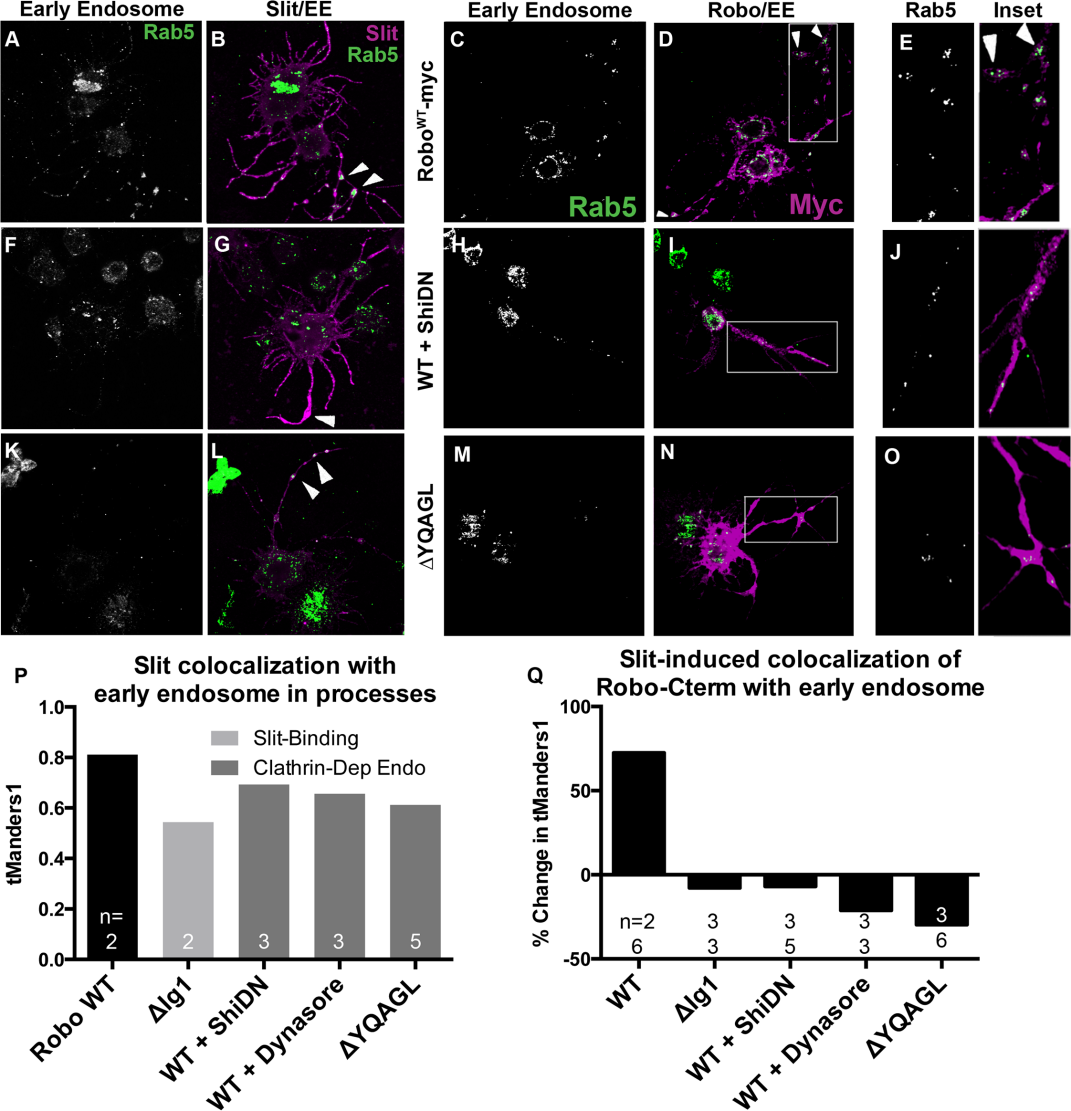
Slit induces Robo colocalization with Rab5 in cell processes. S2R+ cells expressing Robo and treated with SlitCM were fixed at an earlier timepoint (2’) and stained for endogenous Rab5, a marker of the early endosome, (A, C, E, F, H, J, K, M, O) and either bound Slit ligand (B, G, L), or Robo’s C-terminal tag (D, I, N). Cells with Slit bound to processes show covariance between ligand and early endosome signal (B, P (n = # cells indicated on histogram bar)). This colocalization is reduced either by reducing Slit-binding (ΔIg1 in P), or by inhibiting Clathrin-dependent endocytosis globally with DN-Shibire (F, G), or the Dynamin inhibitor Dynasore (P), or by deleting Robo’s AP-2-binding motifs (K, L). Treatment with Slit CM induces colocalization between Robo and Rab5 in processes as compared to cells treated with Control CM, quantified as a percent increase of thresholded Mander’s Overlap Coefficient between Slit and Control CM (C-E, Q). Inhibiting Clathrin-dependent endocytosis by coexpression with DN-Shibire (H-J), use of Dynasore, or deleting AP-2 adaptor motifs (M-O), or inhibiting Slit-binding by deleting the first Ig domain, causes a loss of Slit-dependent colocalization between Robo C-terminus and the early endosome in processes, quantified as the percent change in colocalization between Slit and Control CM (Q). The percentage change switches from positive to negative (Q (n’s for Ctrl CM on top, Slit CM on bottom). See also S4 Fig.

If our receptor manipulations indeed disrupt endocytosis, then we would expect to observe an effect on the intracellular trafficking of internalized Robo in experiments where we track Robo’s C-terminus *in vitro* following Slit treatment. We find that not only do our C-terminal motif deletions inhibit the Slit-dependent removal of Robo from the surface, but they also reduce Slit-dependent colocalization of Robo with endogenous Rab5, a marker of the early endosome. Immunostaining for Slit and Rab5 reveals colocalization between Slit and the early endosome in cell processes (Fig 5A, 5B and 5P). In response to Slit treatment, we also observe an induction of colocalization between Robo and Rab5, specifically in the varicosities and branchpoints of cell processes (Fig 5C–5E), the same structures that showed Slit-dependent surface Robo turnover (arrowheads in Figs 4B and 5B). We have quantified this response as the percentage change in Manders’ overlap coefficient between Slit and Control CM treatment (Fig 5Q). Expression of DN-Shibire (Fig 5F and 5G), or deletion of Robo’s AP-2-binding motifs (Fig 5K and 5L), prevents the Slit-dependent recruitment of Rab5 in cell processes, resulting in less colocalization of Slit with Rab5 (Fig 5P). There is a concomitant reduction in colocalization of Rab5 with the Robo C-terminal tag in the same endocytosis-deficient conditions (Fig 5H–5O and 5Q). These data provide evidence that Slit stimulates the translocation of Robo to the early endosome, and that this process requires Clathrin-dependent endocytosis specifically from the surface of cells.

### Robo endocytosis is required for Sos recruitment

**Fig 6:**
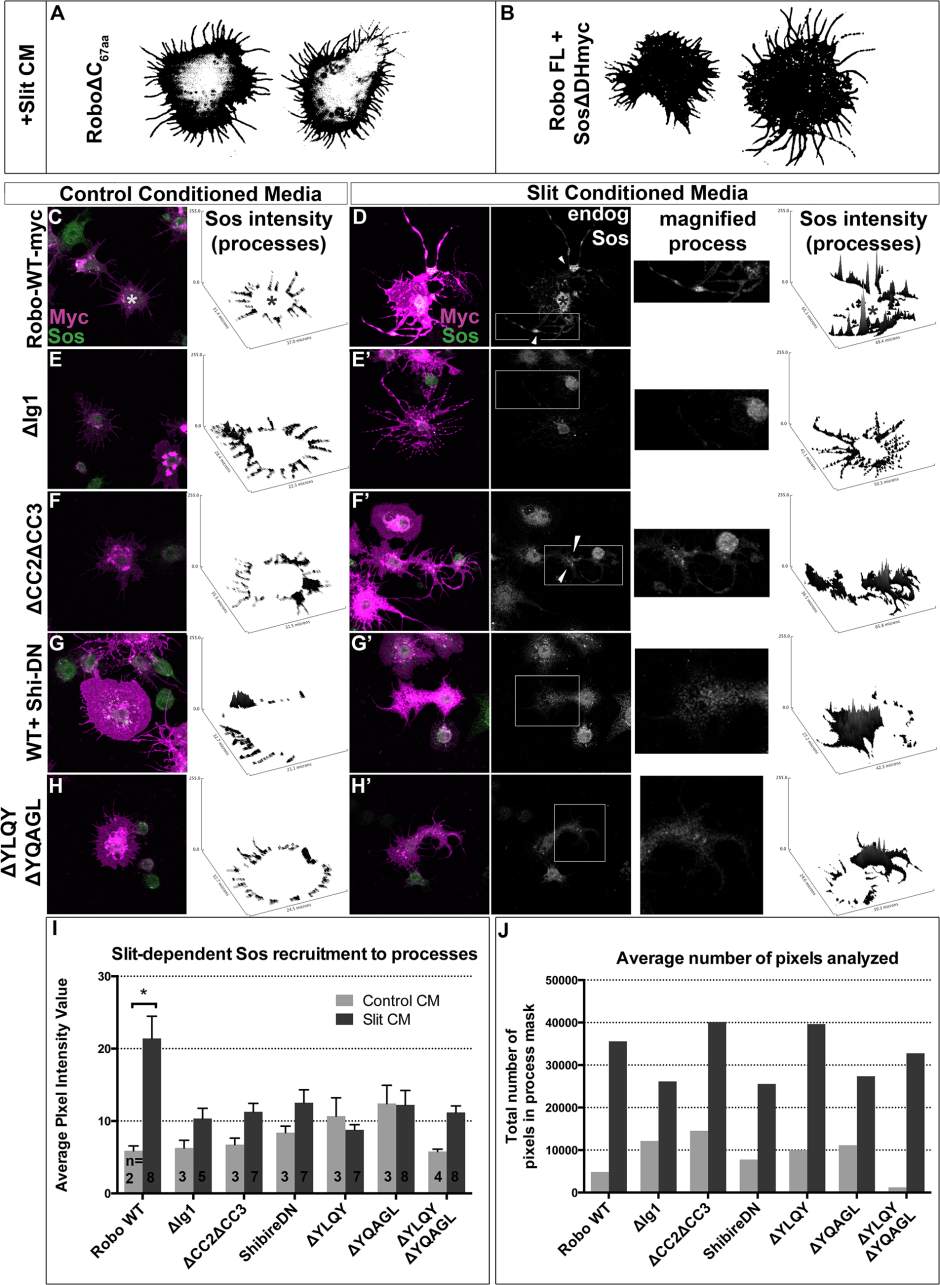
Robo Endocytosis is required for Sos recruitment *in vitro*. Co-expression of a version of Son of Sevenless dominant-negative for its RacGEF activity (B) inhibits spreading and branching of processes in response to Slit CM as effectively as deleting Robo’s entire C-terminus (A). Feature extraction of the pixel intensity of endogenous Sos in processes reveals recruitment of Sos to processes in Slit (D) versus Control CM (C) treatment. The increase in Sos signal in processes in response to Slit seen in Robo^*WT*^-expressing cells, quantified in the histogram as a statistically significant increase (*) in average signal intensity (I, n’s displayed on histogram), is missing in cells expressing RoboΔIg1 (E, E’). Cells expressing a Robo∆CC2∆CC3 receptor that can’t bind Ena or Dock, required for Sos binding (F, F’) also show impaired recruitment of endogenous Sos to processes, as do conditions inhibiting endocytosis (G-H’), despite comparable number of pixels (process area) analyzed (J). Statistical significance quantified by two-way ANOVA, Sidak’s 95% Confidence Interval. Error bars indicate standard error of the mean.

If Robo endocytosis is required for downstream signaling, then we would predict that inhibiting Clathrin-dependent endocytosis of Robo may prevent the recruitment of Son of Sevenless, which has previously been shown to be recruited to Robo in response to Slit-treatment in mammalian cells [48]. First, we assayed the relative contribution of Sos to the spreading behavior in our *in vitro* activation assay by co-expressing Sos missing its RacGEF domain (Fig 6B). This dominant-negative construct blocks the Slit-dependent spreading behavior so effectively that the morphology of these cells are indistinguishable from those expressing Robo missing its entire C-terminus (Fig 6A), indicating that this *in vitro* activation assay depends on the ability of Sos to activate Rac. Having shown that Sos is required for Robo-dependent cell spreading, we sought to examine the capacity of Robo to direct the subcellular localization of endogenous Sos in response to Slit treatment. Extracting the feature of endogenous Sos fluorescence intensity in processes reveals an increase in signal in Slit CM (Fig 6D) over Control CM-treated Robo-expressing cells (Fig 6C and 6I), consistent with recruitment of Sos to processes in response to Slit treatment. Not only is Sos required for process elaboration in response to Slit, and actively recruited into the processes in cells treated with Slit, but it also it is also localized to regions previously shown to carry hallmarks of endocytic activity (reduction in surface receptor levels (Fig 4B) and receptor colocalization with an early endosomal marker (Fig 5E)). Peaks in endogenous Sos signal in Slit CM processes occur at varicosities and branchpoints (arrowheads Fig 6D’ and 6F’), the same structures that are enriched for markers of endocytic activity. Further evidence that Sos recruitment to processes depends on Slit binding comes from the observation that deleting the Ig1 domain or deleting the CC2 and CC3 domains also block Sos recruitment (Fig 6E–6F’). Finally, inhibiting Clathrin-dependent endocytosis also abrogates the increase in endogenous Sos signal intensity in Slit-CM- treated processes over Control CM-treated processes (Fig 6G–6H’), consistent with a model in which Sos recruitment depends on, and therefore occurs following, Clathrin-dependent endocytosis of the Robo receptor in response to Slit-binding.

### Robo endocytosis is required for axon guidance *in vivo*

**Fig 7:**
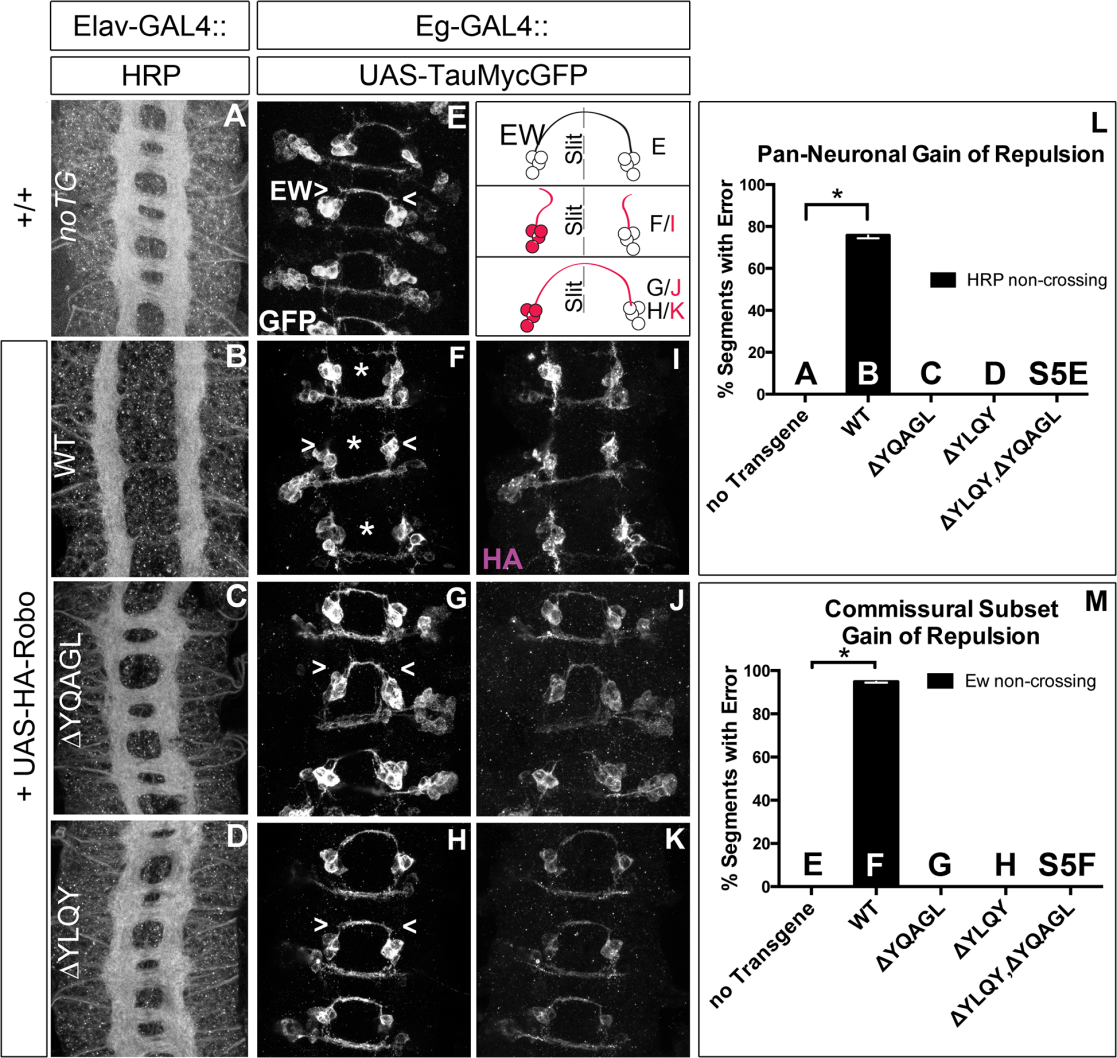
Endocytosis motifs are required for ectopic repulsion *in vivo*. The projection pattern of all axons of the ventral nerve cord of late stage 14 *Drosophila* embryos are imaged with HRP (A-D) and the fascicles of the Eg commissural subset are imaged with a Tau-myc-GFP transgene (E-H). (A) In wild-type embryos, all segments (3 shown here) have two horizontal commissures, which are quantified as 0% of segments with error in the histogram (L n = 88). (B) Overexpressing wild-type Robo transgene in all neurons causes gain of repulsion from midline Slit, resulting in a loss of commissures in 76% of embryonic segments (L, n = 99). In contrast, overexpressing similar levels of Robo transgene that is missing its AP-2 binding motifs (C, D) can not signal ectopic repulsion from the midline, with all segments projecting in a commissural pattern indistinguishable from embryos without transgene (L ΔYQAGL n = 152, ΔYLQY n = 136, ΔYLQYΔYQAGL n = 88). (E) The Ew commissural subset of axons, schematized on the right, cross the midline in each embryonic segment, quantified as 0% error in (M, n = 88). (F) Expressing wild-type Robo transgene (I) specifically in the Ew commissural subset of axons is sufficient to cause ectopic repulsion, with loss of projection across the midline (schematized in dotted gray) in 96% of embryonic segments (M, n = 99). In contrast, expressing either Robo∆YQAGL (G, J) or Robo∆YLQY (H, K) does not cause ectopic repulsion of the Ew projection pattern, with a 0% error in (M, ΔYQAGL n = 152, ΔYLQY n = 136, ΔYLQYΔYQAGL n = 88). Error bars indicate standard error of the mean. See also S5 Fig.

Next, to test whether Robo endocytosis is important for its activation *in vivo*, we assayed these Robo constructs that are defective in Clathrin-dependent endocytosis for their midline guidance activity. First, we overexpressed either wild-type or mutant Robo transgenes in an otherwise wild-type background in two ectopic repulsion assays. All of the transgenes that we used were tagged with an HA epitope, inserted in the same genomic site and were expressed at comparable levels based on immunostaining for their HA epitope tags (Fig 7I–7K). Driving expression of wild-type Robo in all neurons (Fig 7B) is sufficient to signal repulsion so strongly that we see 76% of embryonic segments do not form commissures (Fig 7L). In contrast, none of our endocytosis-defective deletion constructs are able to disrupt midline crossing when similarly expressed (Figs 7C, 7D, 7L and S5E). We see a similar requirement for endocytosis motifs in a commissural subset of axons- the EW axons- whose projection pattern is imaged in Fig 7E with GFP and schematized on the right as a crossed fascicle. Overexpressing wild-type Robo specifically in this subset (Fig 7F and 7I) causes ectopic repulsion from the midline (Fig 7M). In contrast, Robo missing its endocytosis motifs (Figs 7G–7K, 7M, S5F and S5G) does not cause ectopic repulsion, consistent with a requirement for endocytosis of the Robo receptor for its repulsive midline guidance activity *in vivo*. If these AP-2 interaction motifs are indeed required for repulsive signaling then one would predict that over-expressing them might compete with endogenous receptors for access to ligand, thereby acting as a dominant-negative for midline repulsion. Accordingly, in embryos with reduced Slit dosage, expressing a Robo transgene missing both its AP-2 motifs, like that missing its entire C-terminus, does inhibit midline repulsion causing ectopic crossing of the medial-most FasII fascicles (S5B–S5D Fig).

**Fig 8:**
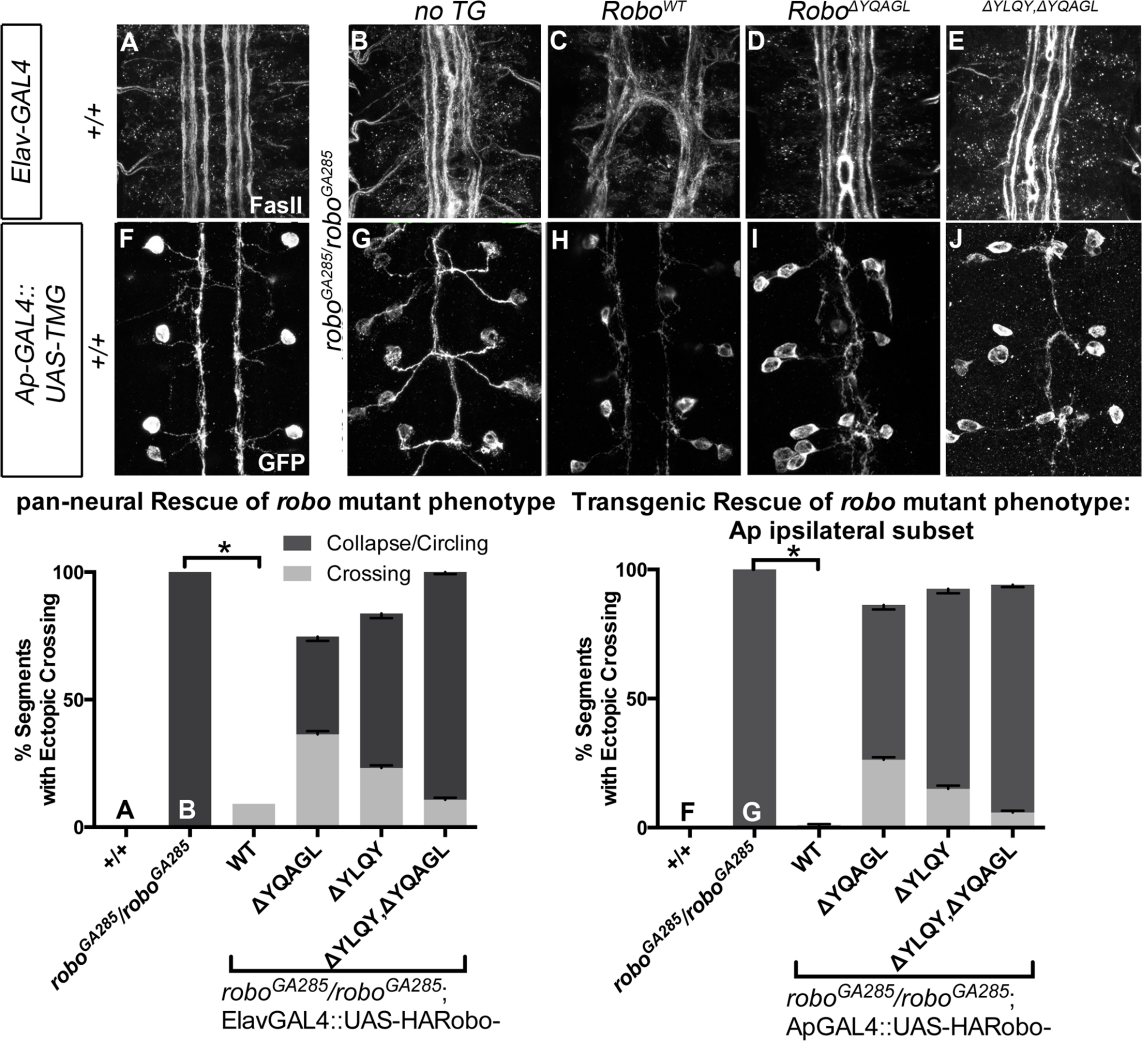
Robo Endocytosis is required for axon guidance *in vivo*. Two ipsilateral subsets of axons are imaged in Stage 17 *Drosophila* embryos- the FasII+ axons with a monoclonal antibody to FasII (A-E) and the Ap axons (F-J) with a GFP antibody detecting Tau-Myc-GFP transgene. In wild-type embryos these ipsilateral subsets project on either side of the midline, with three fascicles on either side for FasII (A) and one fascicle on either side for Ap (F). In *robo* mutant embryos, the two medial-most of the FasII+ fascicles (B) and both of the Ap fascicles (G) collapse onto the midline, scored as 100% of embryonic segments having ectopic collapse/circling events. Expressing wild-type Robo transgene is sufficient to restore repulsive signaling and therefore rescue the crossing defects in the FasII+ axons (C, +/+ n = 121, *robo* ^*GA285*^ */robo* ^*GA285*^ n = 121, *robo* ^*GA285*^ */robo* ^*GA285*^;ElavGAL4/UAS-Robo^*WT*^ n = 121) and the Ap axons (I +/+ n = 120, *robo* ^*GA285*^ */Ap*, *robo* ^*z1772*^ n = 120, *robo* ^*GA285*^ */Ap*, *robo* ^*z1772*^;UAS-RoboWT n = 80). In contrast, expressing Robo∆YQAGL (D, I), Robo∆YLQY, or Robo∆YQAGL ∆YLQY (E, J) is not sufficient to rescue the ectopic crossing events, with a large portion of embryonic segments carrying severe errors (crossing/circling events represented by dark gray) remaining. Dark gray indicates a qualitatively more severe crossing error, light gray indicates a less severe crossing error, with the stacked histogram bar height indicating total % of embryonic segments with loss-of-repulsion errors for each genotype. Error bars indicate standard error of the mean. (*robo* ^*GA285*^ */robo* ^*GA285*^; ElavGAL4/UAS-Robo^*∆YQAGL*^ n = 154, *robo* ^*GA285*^ */robo* ^*GA285*^; ElavGAL4/UAS-Robo^*∆YLQY*^ n = 99, *robo* ^*GA285*^ */robo* ^*GA285*^; ElavGAL4/UAS-Robo^*∆YLQY∆YQAGL*^ n = 121. *robo* ^*GA285*^ */ApGAL4*, *robo* ^*z1772*^;UAS-Robo^*∆YQAGL*^ n = 80, *robo* ^*GA285*^ */ApGAL4*, *robo* ^*z1772*^;UAS-Robo^*∆YLQY*^ n = 120, *robo* ^*GA285*^ */ApGAL4*, *robo* ^*z1772*^;UAS-Robo^*∆YLQY∆YQAGL*^ n = 136.)

Finally, to further assess the *in vivo* repulsive function of these receptor variants, we compared the ability of wild-type versus endocytosis-deficient Robo transgenes to rescue the loss of repulsion defects in *robo* mutant embryos in two normally ipsilateral subsets of axons. The FasII-positive axons project in three (Fig 8A), and the Ap axons project in one fascicle (Fig 8F), on either side of the midline. In *robo* mutants the medial-most pair of FasII, and both Ap, fascicles collapse onto the midline (Fig 8B and 8G). Adding back wild-type Robo transgene either in all neurons or specifically in the Ap subset (Fig 8C and 8H) is sufficient to restore the ipsilateral projection pattern of these axons. In contrast, expressing Robo transgenes missing the AP-2-binding motifs, either singly or together, cannot rescue the midline crossing errors in *robo* mutants when expressed in all neurons (Fig 8D and 8E) or specifically in the Ap ipsilateral subset (Fig 8I and 8J), consistent with a requirement for Robo endocytosis in its repulsive guidance function *in vivo*. We note that deleting Robo’s AP-2 binding motifs more greatly impairs midline guidance activity than process outgrowth in our *in vitro* activation assay, suggesting a functional dissociation in the underlying mechanisms of S2R+ process outgrowth. In the future it will be interesting to determine the effect of these small receptor manipulations on dissociated *Drosophila* growth cone responses to Slit-binding.

## Discussion

In this study, we demonstrate genetic interactions between endocytic pathway components and Slit-Robo signaling consistent with endocytosis positively regulating repulsive midline guidance. Several lines of *in vitro* evidence support the idea that Slit-binding triggers Robo endocytosis and that this event is important for receptor activation and downstream signaling. First, we find that inhibiting Clathrin-dependent endocytosis by manipulating Dynamin, or by deleting putative Clathrin adaptor AP-2 consensus sites on Robo leads to increased surface occupancy of Robo. Second, Slit stimulation of Robo-expressing cells leads to co-localization between Slit, Robo and the early endosome marker Rab5, and manipulations that block endocytosis globally or that specifically block Robo endocytosis, prevent Robo co-localization with Rab5. Third, inhibiting Clathrin-dependent endocytosis and entry into the early and late endosome all inhibit the ability of Robo to induce changes in cell morphology, as well as its ability to recruit the downstream effector Sos. In addition, we present *in vivo* evidence that Robo proteins that lack AP-2 binding motifs are unable to induce ectopic repulsion when expressed in all neurons or in subsets of commissural neurons. Finally, we show that in contrast to wild-type Robo, Robo variants missing their AP-2 binding motifs are unable to rescue the midline crossing defects in *robo* mutant embryos. Taken together, these data strongly support the model that Clathrin-dependent endocytosis of Robo in response to Slit is an important step in transmitting Robo’s repulsive signal across the plasma membrane.

### How does Robo endocytosis contribute to spatially restricted repulsive signaling?

In contexts other than axon guidance, endocytic trafficking has been demonstrated to contribute to receptor signaling by allowing receptor recruitment to specific subcellular compartments. In the case of Wingless [51], Notch [52], EGFR and PVR [53] and VEGFR2 [54], receptor activation is regulated by entry into the early endosome in response to ligand-binding at the surface. Regulation of receptor activation by entry into endocytic compartments can occur by gating spatial access to downstream effectors encountered in signaling complexes–such as Rac or CDC42 in the early endosome [55,56], and MEK1 in the late endosome [57], reviewed in [58]. These observations lend precedent to a model in which endocytic trafficking gates Robo’s spatial access to downstream effectors, such as Sos.

The subcellular localization pattern of Slit, Robo, Rab5 and Sos in our *in vitro* process elaboration assay support this model; Slit and Robo-C terminal tag demarcate- with their peaks in fluorescence intensity- varicosities and nascent branch points along processes at the 2’ early time point (arrowheads in Figs 5B, 5E and S4B) which at 10’ become annexes within branch points (arrowhead in Figs 3A and S4D). Within these enlargements occur correlated valleys in surface Robo signal (arrowhead, Fig 4B) and peaks in markers of both early endosome, Rab5 (arrowheads, Figs 4B, 4E and S4B–S4D) and Sos (arrowheads, Fig 6D). Taking the formation of branchpoints to be the readout of repulsive signaling in the process elaboration assay, we propose that Slit binds to Robo to induce recruitment of both Rab5 and Sos to create what become hubs of endocytosis activity within two minutes, a timepoint previously verified as required for Clathrin-dependent endocytosis in S2R+ cells and in growth cones [59,60]. In this model, Slit binding to the cell is instructing the spatial location of Robo internalization to the early endosome and recruitment of its downstream effector Sos. Consistent with this, when Clathrin-dependent endocytosis is inhibited, Slit binding is intact, but fails to induce the recruitment of Rab5 and therefore there is a correlation between loss of both translocation of Robo from the cell surface to the early endosome, and decreased Sos recruitment.

Our data are consistent with a model in which endocytic trafficking is mechanistically contributing to Robo’s activation by fully or partially gating access to its downstream effector Sos. Evidence from the literature suggests that Sos recruitment might not occur exclusively at the surface of the cell as we had previously reported [31,48], but also in closely apposed early or late endosomal compartments [61]. Sos encodes a Pleckstrin Homology (PH) Domain just C-terminal to the Dbl Homology (DH) domain that is required for both its RacGEF function in Slit/Robo midline guidance in the fly [48], and for *in vitro* Robo activation (Fig 6B). PH domains bind phosphoinositols (PI) of the plasma membrane or small GTPases, and are invariably found adjacent to DH domains, strongly suggesting a functional link between DH and PH activity. In the case of Sos the PH domain has been suggested to act as a mechanical switch to allow initiation of the RacGEF activity of the DH domain upon conversion of a bound PIP2 to PIP3 by PI3Kinase (PI3K) [62]. Phosphoinositides have also been linked to early endosome fusion; Rab5 actively recruits PI3K, which in turn is required for Rab5-mediated conversion of plasma membrane to early endosome [63,64]. It will be interesting to determine whether Sos activation downstream of Robo is gated by PI3K in concert with recruitment to the Rab5-positive early endosome, as this would provide a mechanism by which Robo activation requires Clathrin-mediated endocytosis and Rab5 activity.

### How are *in vitro* process elaboration and branching related to *in vivo* repulsion?

At first glance, the ability of Robo to induce elaboration and branching of cell processes *in vitro* may seem inconsistent with a repulsive output; however, our rescue and gain of function genetic data make a strong case that the signaling output that we observe *in vitro* is critical for repulsion *in vivo*. In addition, there is ample precedent for Slit/Robo signaling to induce branching in both *in vitro* and *in vivo* contexts. For sensory afferents that bifurcate and send collaterals into iterative segments of the spinal cord, uniform Slit treatment induces branching *in vitro* either in suspension cultures of Rat DRGs in collagen gels or bath application to rodent trigeminal neurons [4,65]. The branched morphology of the peripheral arbor of trigeminal projections to the eye requires Slits and Robos [10]. Interestingly, bath application of Slit is sufficient to induce Robo1-dependent growth and branching of dendritic fields of mouse cortical neurons [66], similar to our observations of Slit-induced branching and process growth in S2R+ cells.

### A role for Robo endocytosis in filopodial dynamics?

Since Robo is enriched in growth cone filopodia it is likely that during active migration Robo-containing filopodia would mediate adhesive interactions with Slit in the extracellular matrix. Subsequent Slit-induced filopodial retraction likely requires more than the filopodial dynamics provided by Ena- a Robo effector that is known to localize to the distal tips of filopodia [67,68], since Robo missing its CC2 domain is not fully deficient for midline repulsion [47]. A commonality between our *in vitro* activation assay and previous analyses of growth cone collapse in culture may suggest a possible mechansim. Filopodial contact of a sympathetic growth cone to a retinal neurite is sufficient to initiate an increased rate of growth cone movement- a rapid retraction of an actin-rich structure along the existing axon [69], all while filopodia stay attached, suggesting the existence of a retrograde cue from the filopodial point of contact to more proximal growth cone structures. Similarly, fixed imaging analysis of S2R+ cells in our assay reveals that bath-treatment of Slit CM stimulates the extension and branching of processes over those observed in Control CM. Given that the process elaboration response we observe requires the RacGEF domain of Sos, it is likely that the increased rate of motility implicit in the growth upon Slit treatment is due to alterations in Rac-dependent actin dynamics. Since the process elaboration and branching behavior also requires endocytic trafficking from the cell surface to the late endosome, we can speculate that Robo endocytosis is required to direct the Sos-induced actin motility required for spreading *in vitro*. It is the same receptor manipulations that abrogate Clathrin-dependent endocytosis *in vitro* that lead to impaired repulsive signaling *in vivo*, strongly supporting the idea that Robo endocytosis is required for proper repulsive output in the growth cone, perhaps by allowing the actin-based motility that leads to filopodial retraction and growth cone repulsion. Sequence analysis reveals putative AP-2 binding motifs in human Robo1 (S2J Fig), suggesting conservation of the mechanistic contribution of endocytosis to growth cone navigation all the way to humans, further strengthening the possibility of the importance of this trafficking event.

### What might the relevance of Robo endocytosis be to a migrating growth cone?

Might Slit-binding trigger a similar endocytic trafficking cascade in a growth cone, thereby mobilizing Robo so that it could serve as the retrograde cue informing growth cone behavior from the tips of filopodia? Evidence from others shows that Clathrin-dependent endocytosis exists in the right time and place to play such a role in guidance behavior. First, markers of endocytic compartments, including the early endosome, have been identified in the growth cone [70,71]. If endocytosis serves as a general mechanism for expanding the spatial range of an activated receptor after exposure to ligand on filopodial tips, then we would expect to see examples of correlation between guidance cues trafficking retrogradely and guidance behavior. Endocytosis of guidance molecules in the growth cone has been shown to be initiated both from the base of the growth cone central domain and from the tips of filopodia [23,72]. Retrograde movement of endocytic compartments has been reported in the growth cone and in the case of internalized L1CAM movement occurs at the rate of F-actin retrograde flow [73,74], suggesting that endocytic trafficking could provide an effective spatial track from which a guidance cue might influence the cytoskeleton to affect growth cone behavior. The timing reported by others of endocytosis in the growth cone also shows correlation with the endocytic trafficking of Robo we characterize here *in vitro*. At the same two minute timepoint we report Slit induces Robo removal from S2R+ cell surface here, Sema-3A has affected both a reduction in Neuropilin-1 levels [21,60]–and growth cone collapse in the *Xenopus* RGC growth cone, albeit with different ligand concentrations [75]. Finally, Frizzled endocytosis in a migrating growth cone reveals a correlation between filopodial dynamics and Frizzled endocytosis [23]. It remains to be determined whether retrograde Robo movement from the tips of filopodia is required for repulsion in response to Slit.

Finally, here we have addressed how an endocytic cascade positively contributes to signaling from the Robo receptor, effectively expanding the spatial range of activated receptor within the growth cone. While allowing exposure to the machinery within the growth cone beyond filopodial tips would be required for behaviors such as growth cone retraction or turning in response to filopodial contact with Slit, allowing a receptor to signal too far from the spatial origin of its cue might ultimately prove confusing to a growth cone. It will be interesting to learn if there is a process that serves to curtail signaling from an endocytosed and activated receptor.

## Materials and Methods

### Genetics

The following Drosophila mutant alleles were used: *robo* ^*GA285*^, *robo* ^*z1772*^, *robo* ^*5*^, *slit* ^*1*^, *slit* ^*2*^, *slit* ^*e158*^, *endoA* ^*EP297*^, *endoA* ^*∆4*^, *endoA* ^*10*^, *ada* ^*1*^, *ada* ^*3*^, *rab5* ^*2*^, P[lacW]Rab5^k08232^, P[EPgy2]Rab7^EY10675^, *rab7* ^*FRT82B(*knock-out)^. The following transgenes were used: P[UAS-Shi.K44A]4–1;UAS[*shi*.*K44A]3–7*, P[UASp-YFP-Rab5.S43N], P[UASp-YFP-Rab7.T22N]06, P[UASp-YFP-Rab4.S22N]37, P[UASp-YFP-Rab11.S25N]35. The following transgenic flies were generated by BestGene Inc (Chino Hills, CA) using ΦC31-directed site-specific integration into landing sites at cytological position 86F8 (controlling for expression level effects from chromosomal position): P*[5xUAS-3xHA-Robo-6xmyc]*, *P[5xUAS-3xHA-Robo* ^*∆YLQY-*^ *6xmyc]*, *P[5xUAS-3xHA-Robo-1xmCherry]*, *P[5xUAS-3xHA-Robo* ^*∆YQAGL*^ *-1xmCherry]*, *P[5xUAS-3xHA-Robo* ^*∆YQAGL*^ *-6xmyc]*, *P[5xUAS-3xHA-Robo* ^*∆YLQY∆YQAGL*^ *-6xmyc]*, *P[10xUAS-3xHA-Robo* ^*∆Ig1*^ *]*, *P[10xUAS-3xHA-Robo* ^*∆C*^ *-6xmyc]*, *P[10xUAS-3xHA-Robo* ^*∆YLQY∆YQAGL*^ *-6xmyc]*. Also used were the extant lines *P[GAL4-elav*.*L]3 (elav-GAL4)*, *eg* ^*MZ360*^ (*eg-GAL4)*, *ap*-*GAL4*. Embryos were genotyped using balancer chromosomes carrying *lacZ* markers or by the presence of epitope-tagged transgenes.

### Molecular biology

#### pUAST cloning

Robo coding sequences were cloned into a pUAST vector (p5UASTAttB) including 5xUAS and an attB site for ΦC31-directed site-specific integration. All p5UASTattB constructs include identical heterologous 5’ UTR and signal sequences (derived from the Drosophila wingless gene) and an N-terminal 3×HA tag. Robo domain deletion variants created for this study were generated by PCR and include the following amino acids (numbers refer to Genbank reference sequences AAF46887 [Robo]: Robo^∆Ig1^ (153–1395), Robo^∆C^ (56–950), Robo^*∆YLQY*^ (1090–1093), Robo^*∆YQAGL*^ (1233–1237), Robo^*∆YLQY ∆YQAGL*^ (1090–1093; 1233–1237), Robo^*Y1090A*^ (1090), Robo^*Y1233A*^ (1233), Robo^*Y1090A*,*Y1233A*^ *(1090;1233)*. All Robo constructs used in the *in vitro* S2R+ activation assay were cloned into the 5xUAS-AttB plasmid containing 3xHA(N) and 6xMyc(C) tags, or 3xHA no C-terminal Tag (Barry Dickson, shuttled into p5AttB here), or 3xHA-1xpHluorin: The pHluorin-Robo tag was added with the following primers: TAGCTAGCAGCAAAGGAgAAGAAc, CGATCGAGATCCGGAGCTAGCTA; 1x-mCherry C-terminal tag was obtained by PCR amplification of mCherry CDS genomic extraction of mCherry::CAAX flies (Kyoto DGRC courtesy of Roger Tsien) using the following primers: at**actagt**atggtgagcaagggc, atatata**gcggccgc**TTActtgtacagctcgtcca to swap out the 6xMyc tag using SpeI/NotI sites; or deletion of the 3xHA tag to include 6xMyc tag only by first deleting the BmtI sites in the backbone using the following primers: AAATGCTTGGATTTCACTGGAACTAGGCTTTC

ATAACTTCGTATAATGTATGCTATACGAAGTTATGCTAGCG,CGCTAGCATAACTTCGTATAGCATACATTATACGAAGTTATGAAAGCCTAGTTCCAGTGAAATCCAAGCATT,GGCTTTCATAACTTCGTATAATGTATGCTATACGAAGTTATGCTTTCGGATCCAAGCTGGCCG,CGGCCAGCTTGGATCCGAAAGCATAACTTCGTATAGCATACATTATACGAAGTTATGAAAGCC, (also the template for ΔIg1 in p5AttB) then serial overlap extension PCR with the following primers: tatatata**GAATTC**TATCATACCCCGTGTGTCAGTGTG,GCTCGATGATACGTGGATCTAA**GCTAGC**GCGCGCCCTTCCGGAT,ATCCGGAAGGGCGCGCGCTAGCTTAGATCCACGTATCATCGAGC, GTTTGATTGGCAGGTCCGATTTGAA. Robo ΔEcto (3xHA/6xMyc) was created by PCR using the following primers: tatataCGCTAGCatg**ACCACTGACTACCTATCTGGACC,** tcgggtggctattgggatgc. RoboΔYLQY was created using the following primers: TTGTCAAATCCAAC**CCGGTTGAACCGATCA,**TGATCGGTTCAACCGG**GTTGGATTTGACAA**; ALQY: GTCAAATCCAAC**gcc**CTTCAGTATCCG, CGGATACTGAAGGGCGTTGGATTTGAC; ΔYQAGL: CAGCCAGCGAG**AATGCAGCG,** CGCTGCATTCTCGCTGGCTG; AQAGL: CAGCCAGCGAG**gcCCAGGCT,** AGCCTGGgcCTCGCTGGCTG;

#### pALG cloning

*WT* Rab5 was obtained from Dr. Avital Rodal in the Actin5C promoter N-terminal GFP-tag pALG plasmid. The Rab5 Dominant-Negative (DN) S43N point mutation was created with the following primers:CGAGTCCGCTGTGGGCAAGAACTCACTGGTGCTGCGCTTCG, CGAAGCGCAGCACCAGTGAGTTCTTGCCCACAGCGGACTCG. *WT* Rab7 was obtained from BDGP Gold Collection (clone ID GH03685) and shuttled into pALG using the following primers:**tatataGCGGCCGCCCCCTTCACCATG**ATGTCCGGACGTAAGAAATCCCTACTGAA, **TATATATAcgatcg**TTAGCACTGACAGTTGTCAGGATTGCC. The Rab7 T22N point mutation was created by PCR with the following primers:TGTGGGCAAGAACTCTCTGATGAAT, GATTCATCAGAGAGTTCTTGCCCACACT. *shibire* cDNA was PCR amplified from pOT2 BDGP Gold Collection clone (LD21622) and shuttled to pALG using the following primers: **ataGCGGCCGCCCCCTTCACCATG**atggatagtttaattacaattgttaacaagctgcaa,**TATATATAcgatcg**attacttgaatcgcgaactgaaggcat. Shibire K44A (DN) was created using the following primers: gaactttgtgggc**GC**agatttcttgcc, **ggcaagaaatctGCgcccacaaagttc.**

### Immunofluorescence and imaging

#### *In vitro* Robo activation assay

*Drosophila* S2R+ cells were cultured at 25°C in Schneider’s media plus 10% FBS and 1% Penicillin-Streptomycin. To assay for Slit response, cells were plated on acid-etched, poly-L-lysine coated coverslips in duplicate in six-well plates (Robo-expressing cells) at a density of 1–2×10^6^ cells/mL, and transfected with 0.25ug of p5AttB construct and pMT-GAL4/2mL Schneider’s (a one-day lag between CM and Robo cells) using Effectene transfection reagent (Qiagen). GAL4 expression was induced with 0.5 mM CuSO_4_ for 24 hours, then Slit-Conditioned Media (CM) was collected and concentrated (Amicon Ultracel 30K) from cells transfected with empty pUAST vector or Slit. Robo-transfected cells were incubated with CM on an orbital shaker at room temperature for 2 (pHluorin, Rab5 colocalization, Sos recruitment) or 10 minutes (process area/Sholl analysis), then fixed for 10 minutes at RT in 4% PFA. Cells were rinsed with 1XPBS, permeabilized with PBS+0.1% Triton X-100 (PBT) for 2 minutes, then blocked for 1Hr and stained with antibodies diluted in PBT+4% NGS, except for the pHluorin surface assay, which used no detergent and MetOH-free PFA. Antibodies used were: mouse anti-Slit-C (c555.6D, DSHB, 1:100), mouse anti-cMyc (9E10, 1:1000), rabbit anti-cMyc (Sigma c3956, 1:1000), rabbit anti-GFP (Invitrogen #A11122, 1:1000), rabbit anti-HA (Covance, 1:1000), rabbit anti-*d*Rab5 (abcam 31261,1:1000), rabbit anti-Sos (SantaCruz C23 1:500), Cy3 goat anti-mouse (Jackson Immunoresearch, 1:1000), and Alexa488 goat anti-rabbit (Molecular Probes, 1:500). Coverslips were mounted in Aquamount. 0.252μM totalZ confocal stacks were collected using a Leica TCS SP5 confocal microscope at 63X and zoom3. Representative S2R+ cells were selected for imaging while blinded to transfection condition.

#### Quantification

The morphological profiles of S2R+ cells were generated by binary thresholding the signal from Slit or Myc (Robo-Cterm epitope tag), then we manually defined a region of interest cropping out the variably sized cell cortices and computed for **process area** the total pixel area of the process (FIJI histogram: count-mode), and averaged across many cells for each condition to compute average process area. For the **Sholl analysis** a manually drawn ray defined the center of the cell with a starting radius manually defined starting after the cell cortex and continuing until the maximal radius of the process field (with any intervening cells cleared from the background) was entered into FIJI Sholl plugin. The resulting number of intersections from multiple cells were registered onto a common radius scale in Excel and the average number of intersections for each radius/genotype was plotted, only for the radii for which more than one cell had process field. For **colocalization analysis**, signal subtraction was applied across all images: Myc-80, Rab5-15 (Slit-0), a region of interest around the processes was defined, then the average Mander’s Overlap Coefficient was computed from multiple representative cells (n’s indicated in histograms) from FIJI Coloc2 algorithm in the Slit-only condition for Slit-Rab5, or the ratio of SlitCM/Control CM for RoboMyc-Rab5. For **pHluorin intensity** the average GFP signal intensity was isolated from background by computing the average pixel intensity value, from a manually defined ROI around processes only, of values above intensity4 (determined empirically to be maximum value of ‘black’ cell-negative regions).

#### Embryos

Dechorionated, formaldehyde-fixed, methanol-devitellinized *Drosophila* embryos were fluorescently stained using standard methods. The following antibodies were used in this study: FITC-conjugated goat anti-HRP (Jackson # 123-095-021, 1:250), mouse anti-Fasciclin-II/mAb 1D4 [Developmental Studies Hybridoma Bank, (DSHB), 1:100], mouse anti-βgal (DSHB, 1:150), Alexa-488 conjugated goat-anti-HRP (Jackson #123-605-021 1:100), Cy3-conjugated goat anti-mouse (Jackson #115-165-003, 1:1000), Alexa-488-conjugated goat anti-rabbit (Molecular Probes #A11008, 1:500). Embryos were filleted and mounted in 70%glycerol/1XPBS and imaged on Leica TCS SP5 at 63X with a zoom of 1.7. Images were processed using FIJI.

### Biochemistry

Control and Slit CM were boiled for 10’ in 2X SDS Loading Buffer. Proteins were resolved by SDS Page and transferred to nitrocellulose and incubated with anti-Slit-C (C555-6D) 1:100 overnight at 4°C in PBS/0.05% Tween-20/5% non-fat dry milk. Blots were incubated with HRP-conjugated anti-mouse secondary antibody for 1 hour at RT and signal was detected using ECL Prime (Amersham).

## Supporting Information

S1 Fig Endocytosis positively regulates Slit-Robo repulsion, not Frazzled-mediated attraction. A: Expression of UAS-HA-Robo transgenes specifically in the Ap subset of neurons rescues the defects caused by overexpression of ShibireDN and Rab5DN in *slit* ^*2*^ */+* heterozygotes. B: Reducing the dosage of endocytic trafficking genes do not enhance (not statistically significant, n.s.) the ectopic crossing errors induced by enhanced midline attraction resulting from ectopic expression of the attractive guidance receptor Frazzled beyond the predicted percentage crossing frequency from an additive interaction in the Ap neurons. Error bars indicate standard error of the mean.
(TIF)

S2 Fig Endocytosis positively regulates Robo signaling *in vitro*. Representative examples of the morphological profiles of Drosophila embryonic cells transfected with Robo and bath-treated for 10’ with Control CM or Slit CM are displayed. (A) Cells that express Robo deficient for Slit-binding by deletion of the first Ig domain, or by deletion of the entire ectodomain (A’, schematized in domain structure cartoon below) do not elaborate processes as much (A) or at all (A’) in response to Slit treatment. (B) Western blot of control and Slit-conditioned media probed with an antibody against Slit. (C) Robo missing its CC2 and CC3 domains, required for Rac activation, display a qualitatively distinct class of impaired process elaboration. There is an increase in the number of short branches, but the total process area and therefore Sholl profile is shunted as compared to WT-expressing cells (Sholl n = 11). Inhibiting Clathrin-dependent endocytosis either directly by treatment with 20μM Dynasore, a dynamin inhibitor (D, n = 5), or by deleting its AP-2 binding motifs together (E), or point-mutating the catalytic tyrosines each singly (F, G) or both together (H) leads to the same qualitative type of spreading behavior. In all cases, there is a reduction in process branching, which is quantified as a reduction in the process area in Slit-CM treated cells and a downward shift in the Sholl profile (quantified on the right) as compared to WT-expressing cells. Deleting both AP-2 motifs together results in a smaller maximal process radius. (I) Expression of the attractive guidance receptor Frazzled causes S2R+ cells to spread in a qualitatively distinct manner with more lamellipodial-appearing spreading that does not respond to Slit-CM treatment. (J) Box-shade alignments of amino acid sequence of the identified AP-2 adaptor motifs show sequence conservation between *Drosophila* Robo1 and Human Robo1, and the originating sequences, suggesting conservation of function throughout phylogeny. Domain structure diagrams show the location of the putative AP-2-binding motifs, and the other variants assayed here, within *Drosophila* Robo1.
(TIF)

S3 Fig Slit-dependent Robo endocytosis occurs upstream of Sos recruitment. A-F: Surface Robo signal in S2R+ cells is isolated by a pH sensitive tag, pHluorin, on Robo’s ectodomain. In cells treated with the Dynamin inhibitor Dynasore, Robo is still expressed on the tips of processes in Control CM conditions (arrowheads, A), but the downregulation of surface signal in response to Slit treatment seen with WT-Robo expressing cells is blunted and surface Robo remains high (B, G). Disrupting Robo’s AP-2 binding motifs singly does not affect the average pHluorin signal intensity in Control CM conditions (C, E), but inhibits the reduction in Slit CM conditions (D, F) seen in WT-Robo expressing cells, resulting in a reduced % decrease in average signal intensity (G). Point-mutating the catalytic tyrosines of the AP-2 motifs singly or together also reduces the Slit-dependent decrease in surface Robo signal, while inhibiting Sos-mediated activation of Rac by co-expressing Son-of-sevenless missing its Dbl Homology domain does not affect surface Robo levels (G). Number of cells analyzed are indicated on the histogram (top number, n from Control CM, bottom number, Slit CM). (H) Signal from an HA epitope tag on Robo’s ectodomain expressed in the ipsilateral Ap subset of neurons is imaged by immunostaining in Stage 16 Drosophila embryos. Inhibiting Slit by either creating slitmutant/hypomorph embryos or by deleting Robo’s Slit-binding domain (∆Ig1) causes mislocalization of Robo to ectopically collapsed or crossing portions of axons, respectively.
(TIF)

S4 Fig Slit colocalization with Rab5 persists over 10’ *in vitro*. Endogenous Rab5 in S2R+ Robo-expressing cells bath-treated with Control CM (A) or Slit CM for 2’ (B), 5’ (C) or 10’ (D). Slit antibody staining is specific for cells treated with Slit CM (B-D), and Rab5 is recruited to processes in cells that have bound Slit. At all three timepoints Slit and Rab5 are colocalized in varicosities and branchpoints of elaborating processes (arrowheads, B-D).
(TIF)

S5 Fig Robo missing its AP2 motifs functions as a dominant-negative *in vivo*. In *slit* ^*2*^ */+* embryos, Robo missing its entire C-terminus (C) functions as a strong dominant-negative for midline repulsion, inducing an 100% error rate (D) when driven in all neurons, quantified here in the normally-ipsilateral medialmost FasII+ axons (A). Like Robo∆C Robo^ΔYQAGLΔYLQY^ overexpression in all neurons in a partial loss of Slit background causes ectopic crossing (B). (E-G) Robo∆YQAGL∆YLQY can not signal repulsion to cause loss of commissural projection pattern when overexpressed either in all neurons (E), or in the Eg commissural subset (F, G).
(TIF)

S6 Fig A schematic model of Robo signaling events. In response to Slit binding, endocytosis of the Robo receptor is followed by the recruitment of the Ras/Rho GEF Son of Sevenless to the cytoplasmic domain of Robo. Sos recruitment results in elevation of Rac activity and repulsive signaling as the receptor trafficks from the plasma membrane to the early and late endosomes.
(TIF)
