# Supplementary figures and images for: Slit-Dependent Endocytic Trafficking of the Robo Receptor Is Required for Son of Sevenless Recruitment and Midline Axon Repulsion
Source: PLoS Genet. 2015 Sep 3;11(9):e1005402. doi: 10.1371/journal.pgen.1005402 (PMC4559387; doi:10.1371/journal.pgen.1005402)

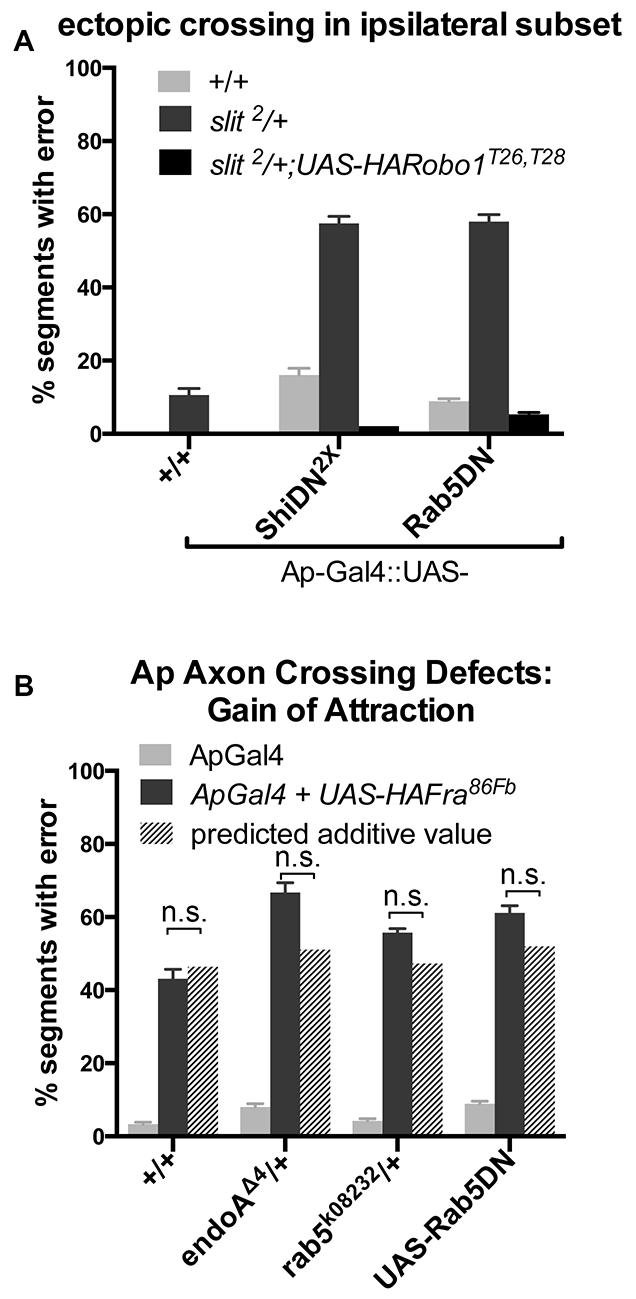

Supplement: S1 Fig — A: Expression of UAS-HA-Robo transgenes specifically in the Ap subset of neurons rescues the defects caused by overexpression of ShibireDN and Rab5DN in slit 2 /+ heterozygotes. B: Reducing the dosage of endocytic trafficking genes do not enhance (not statistically significant, n.s.) the ectopic crossing errors induced by enhanced midline attraction resulting from ectopic expression of the attractive guidance receptor Frazzled beyond the predicted percentage crossing frequency from an additive interaction in the Ap neurons. Error bars indicate standard error of the mean. (TIF) [file pgen.1005402.s001.tif]

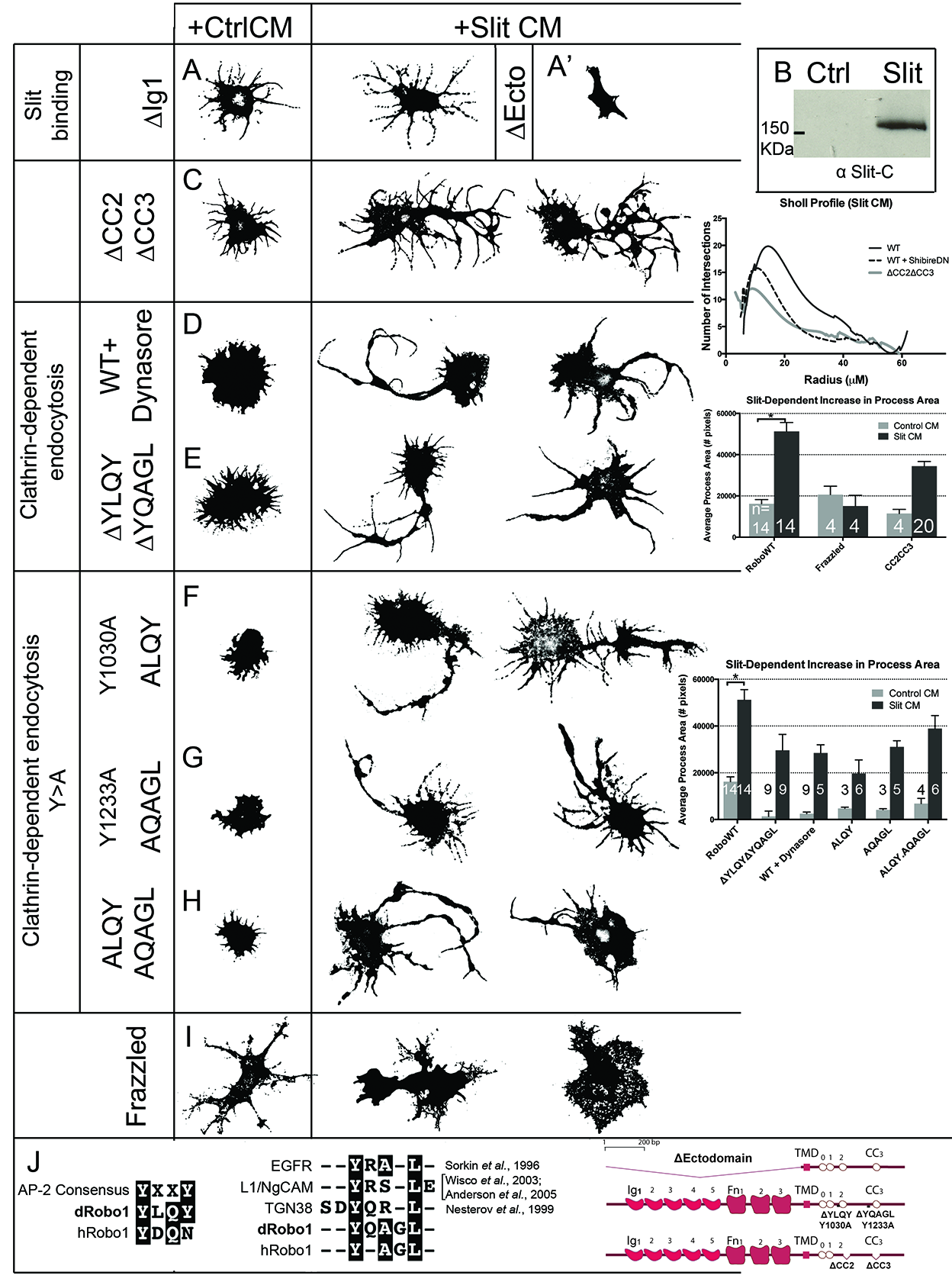

Supplement: S2 Fig — Representative examples of the morphological profiles of Drosophila embryonic cells transfected with Robo and bath-treated for 10’ with Control CM or Slit CM are displayed. (A) Cells that express Robo deficient for Slit-binding by deletion of the first Ig domain, or by deletion of the entire ectodomain (A’, schematized in domain structure cartoon below) do not elaborate processes as much (A) or at all (A’) in response to Slit treatment. (B) Western blot of control and Slit-conditioned media probed with an antibody against Slit. (C) Robo missing its CC2 and CC3 domains, required for Rac activation, display a qualitatively distinct class of impaired process elaboration. There is an increase in the number of short branches, but the total process area and therefore Sholl profile is shunted as compared to WT-expressing cells (Sholl n = 11). Inhibiting Clathrin-dependent endocytosis either directly by treatment with 20μM Dynasore, a dynamin inhibitor (D, n = 5), or by deleting its AP-2 binding motifs together (E), or point-mutating the catalytic tyrosines each singly (F, G) or both together (H) leads to the same qualitative type of spreading behavior. In all cases, there is a reduction in process branching, which is quantified as a reduction in the process area in Slit-CM treated cells and a downward shift in the Sholl profile (quantified on the right) as compared to WT-expressing cells. Deleting both AP-2 motifs together results in a smaller maximal process radius. (I) Expression of the attractive guidance receptor Frazzled causes S2R+ cells to spread in a qualitatively distinct manner with more lamellipodial-appearing spreading that does not respond to Slit-CM treatment. (J) Box-shade alignments of amino acid sequence of the identified AP-2 adaptor motifs show sequence conservation between Drosophila Robo1 and Human Robo1, and the originating sequences, suggesting conservation of function throughout phylogeny. Domain structure diagrams show the location of the putative [file pgen.1005402.s002.tif]

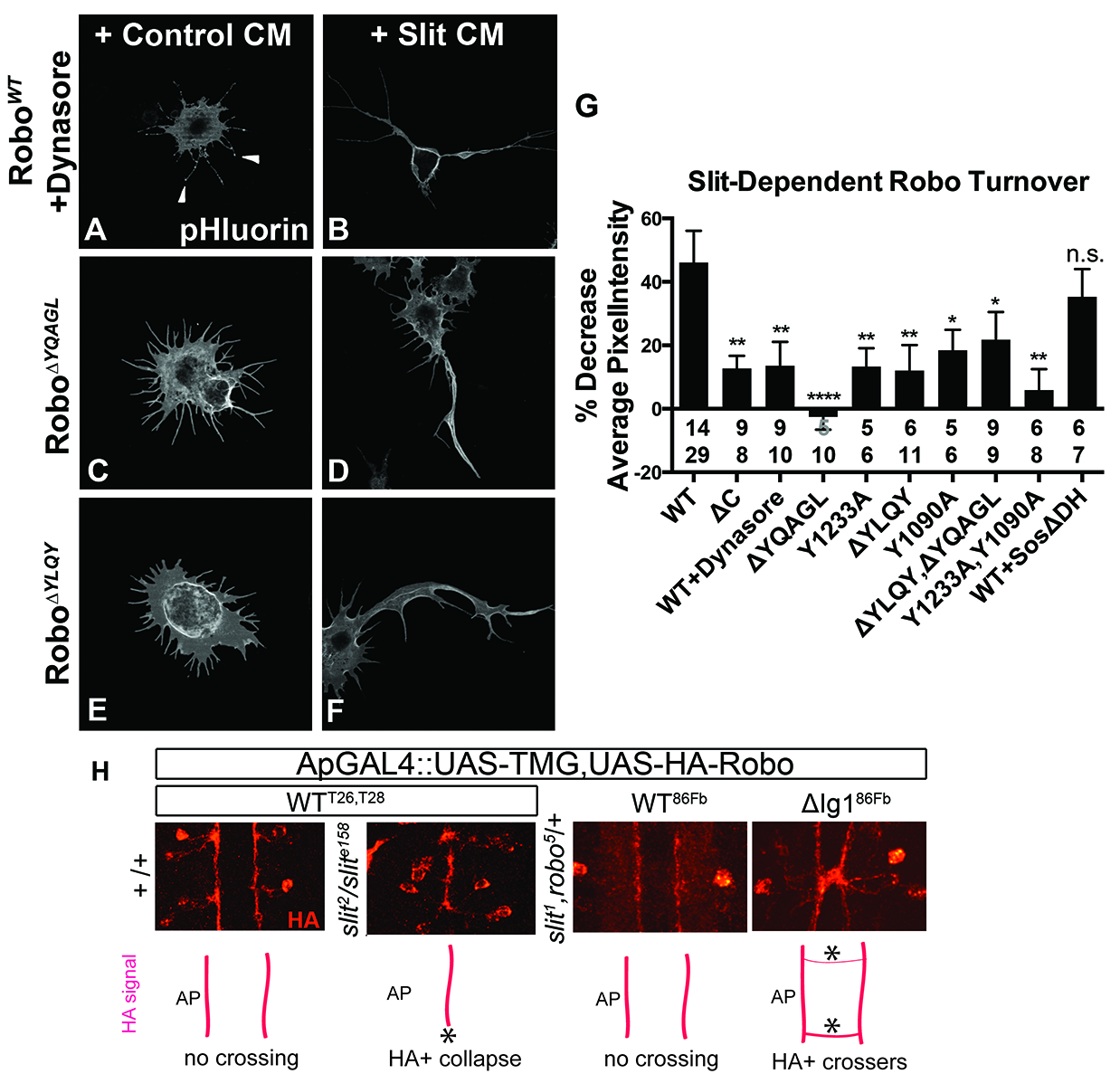

Supplement: S3 Fig — A-F: Surface Robo signal in S2R+ cells is isolated by a pH sensitive tag, pHluorin, on Robo’s ectodomain. In cells treated with the Dynamin inhibitor Dynasore, Robo is still expressed on the tips of processes in Control CM conditions (arrowheads, A), but the downregulation of surface signal in response to Slit treatment seen with WT-Robo expressing cells is blunted and surface Robo remains high (B, G). Disrupting Robo’s AP-2 binding motifs singly does not affect the average pHluorin signal intensity in Control CM conditions (C, E), but inhibits the reduction in Slit CM conditions (D, F) seen in WT-Robo expressing cells, resulting in a reduced % decrease in average signal intensity (G). Point-mutating the catalytic tyrosines of the AP-2 motifs singly or together also reduces the Slit-dependent decrease in surface Robo signal, while inhibiting Sos-mediated activation of Rac by co-expressing Son-of-sevenless missing its Dbl Homology domain does not affect surface Robo levels (G). Number of cells analyzed are indicated on the histogram (top number, n from Control CM, bottom number, Slit CM). (H) Signal from an HA epitope tag on Robo’s ectodomain expressed in the ipsilateral Ap subset of neurons is imaged by immunostaining in Stage 16 Drosophila embryos. Inhibiting Slit by either creating slitmutant/hypomorph embryos or by deleting Robo’s Slit-binding domain (∆Ig1) causes mislocalization of Robo to ectopically collapsed or crossing portions of axons, respectively. (TIF) [file pgen.1005402.s003.tif]

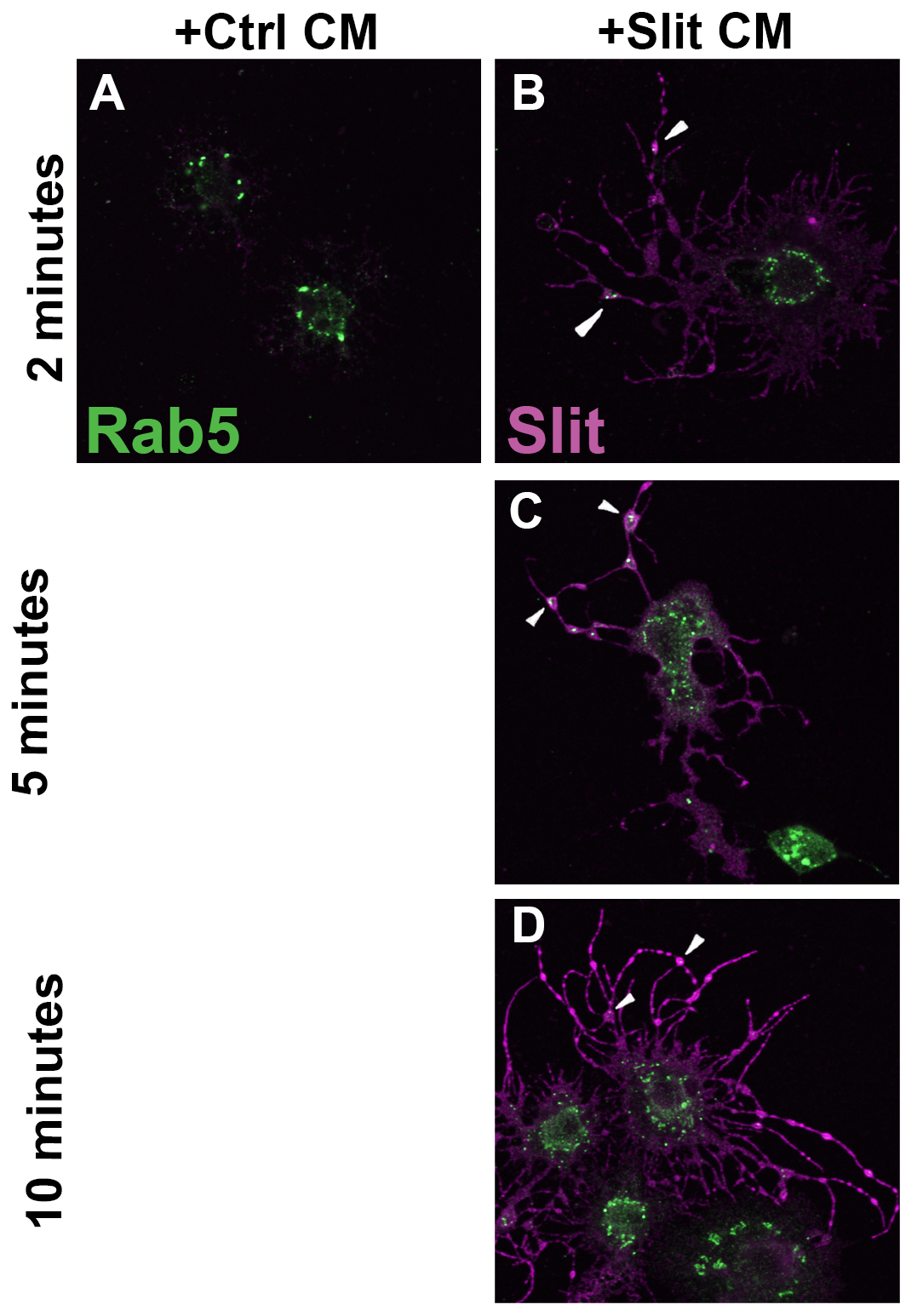

Supplement: S4 Fig — Endogenous Rab5 in S2R+ Robo-expressing cells bath-treated with Control CM (A) or Slit CM for 2’ (B), 5’ (C) or 10’ (D). Slit antibody staining is specific for cells treated with Slit CM (B-D), and Rab5 is recruited to processes in cells that have bound Slit. At all three timepoints Slit and Rab5 are colocalized in varicosities and branchpoints of elaborating processes (arrowheads, B-D). (TIF) [file pgen.1005402.s004.tif]

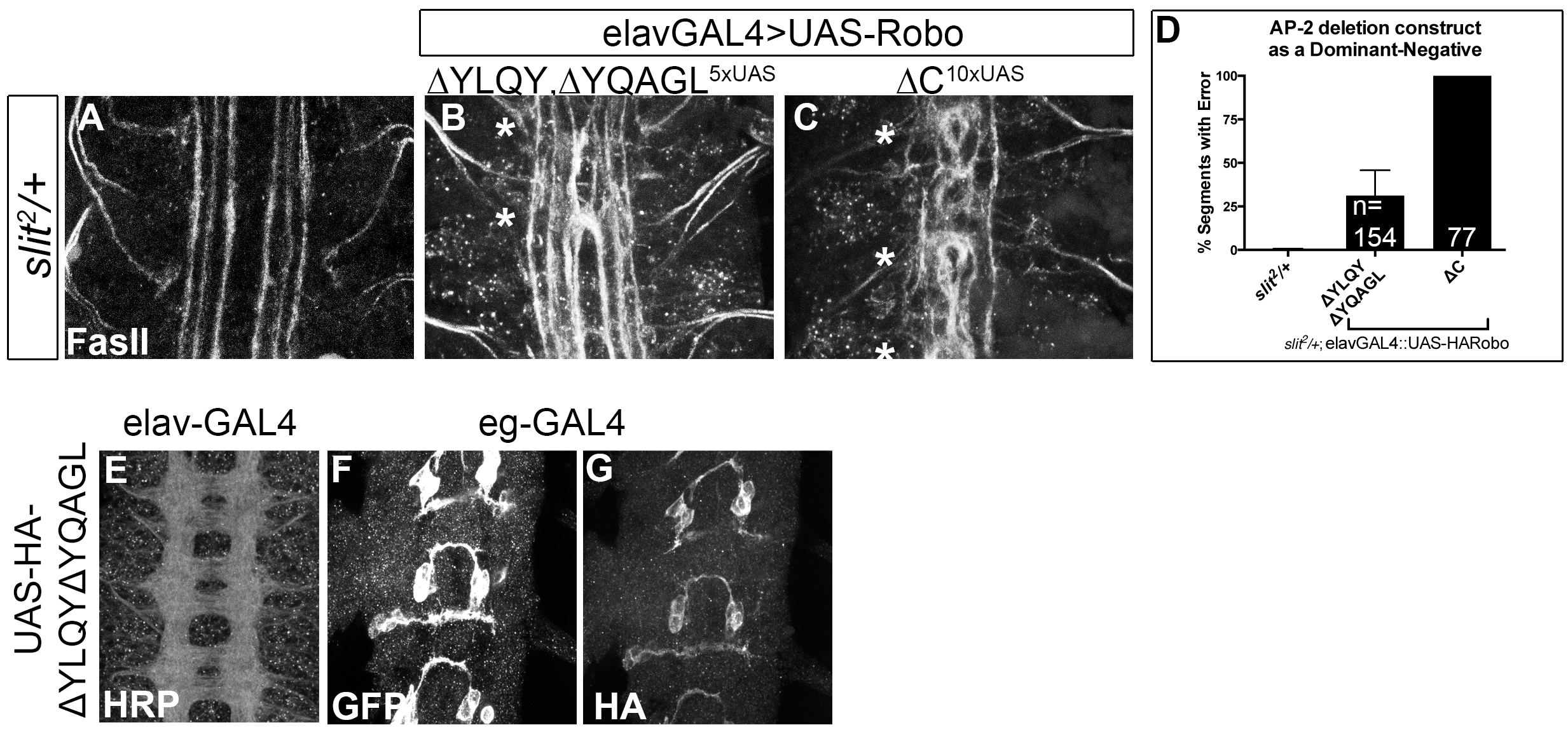

Supplement: S5 Fig — In slit 2 /+ embryos, Robo missing its entire C-terminus (C) functions as a strong dominant-negative for midline repulsion, inducing an 100% error rate (D) when driven in all neurons, quantified here in the normally-ipsilateral medialmost FasII+ axons (A). Like Robo∆C RoboΔYQAGLΔYLQY overexpression in all neurons in a partial loss of Slit background causes ectopic crossing (B). (E-G) Robo∆YQAGL∆YLQY can not signal repulsion to cause loss of commissural projection pattern when overexpressed either in all neurons (E), or in the Eg commissural subset (F, G). (TIF) [file pgen.1005402.s005.tif]

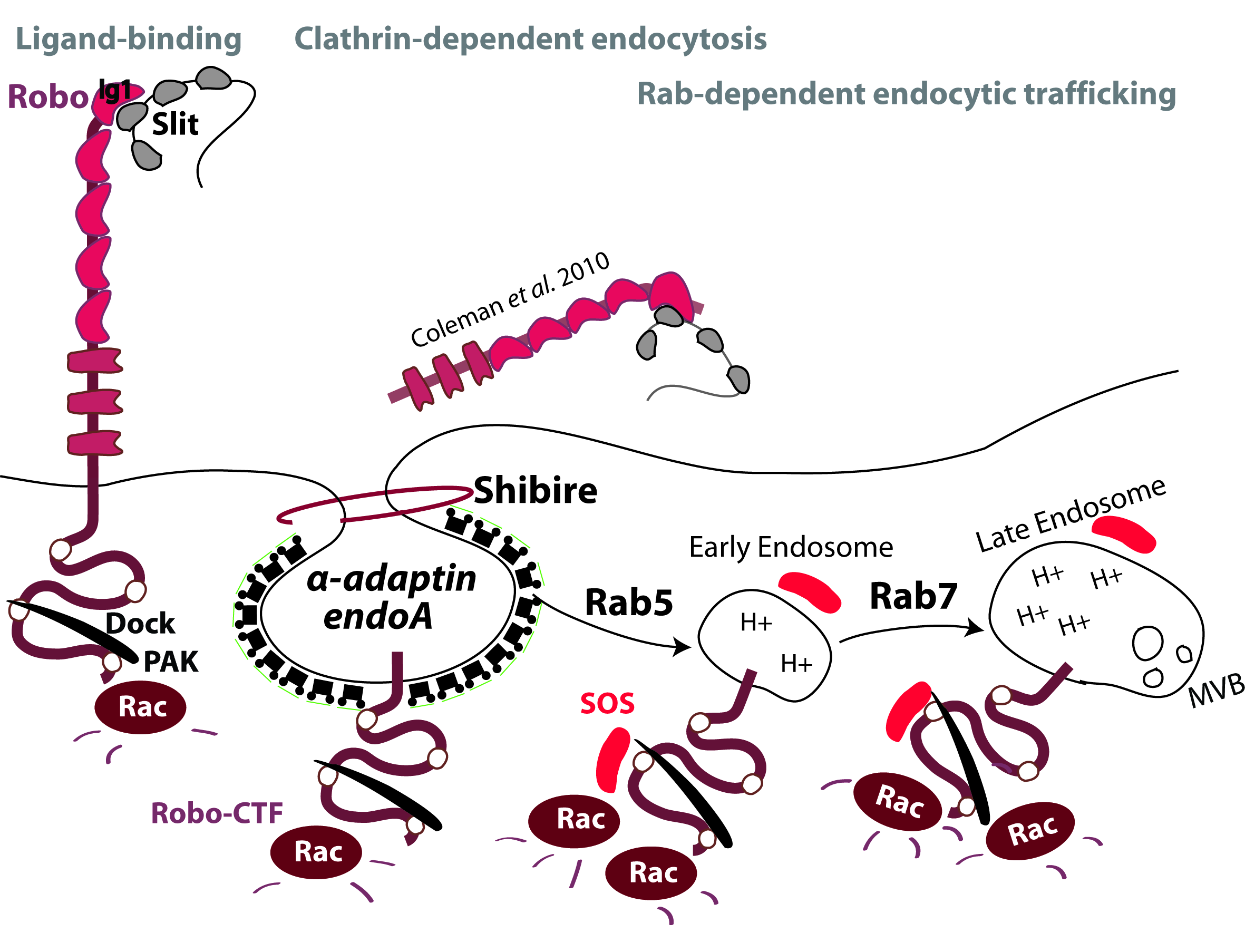

Supplement: S6 Fig — In response to Slit binding, endocytosis of the Robo receptor is followed by the recruitment of the Ras/Rho GEF Son of Sevenless to the cytoplasmic domain of Robo. Sos recruitment results in elevation of Rac activity and repulsive signaling as the receptor trafficks from the plasma membrane to the early and late endosomes. (TIF) [file pgen.1005402.s006.tif]
